# Plasmonic metasurfaces with 42.3% transmission efficiency in the visible

**DOI:** 10.1038/s41377-019-0164-8

**Published:** 2019-06-12

**Authors:** Jihua Zhang, Mohamed ElKabbash, Ran Wei, Subhash C. Singh, Billy Lam, Chunlei Guo

**Affiliations:** 1 The Institute of Optics, University of Rochester, Rochester, NY 14627 USA; 2 Changchun Institute of Optics, Fine Mechanics, and Physics, Chinese Academy of Sciences, 130033 Changchun, China

**Keywords:** Metamaterials, Nanophotonics and plasmonics, Nanophotonics and plasmonics

## Abstract

Metasurfaces are two-dimensional nanoantenna arrays that can control the propagation of light at will. In particular, plasmonic metasurfaces feature ultrathin thicknesses, ease of fabrication, field confinement beyond the diffraction limit, superior nonlinear properties, and ultrafast performances. However, the technological relevance of plasmonic metasurfaces operating in the transmission mode at optical frequencies is questionable due to their limited efficiency. The state-of-the-art efficiency of geometric plasmonic metasurfaces at visible and near-infrared frequencies, for example, is ≤10%. Here, we report a multipole-interference-based transmission-type geometric plasmonic metasurface with a polarization conversion efficiency that reaches 42.3% at 744 nm, over 400% increase over the state of the art. The efficiency is augmented by breaking the scattering symmetry due to simultaneously approaching the generalized Kerker condition for two orthogonal polarizations. In addition, the design of the metasurface proposed in this study introduces an air gap between the antennas and the surrounding media that confines the field within the gap, which mitigates the crosstalk between meta-atoms and minimizes metallic absorption. The proposed metasurface is broadband, versatile, easy to fabricate, and highly tolerant to fabrication errors. We highlight the technological relevance of our plasmonic metasurface by demonstrating a transmission-type beam deflector and hologram with record efficiencies.

## A new class of plasmonic metasurfaces for enhanced light transmission

Researchers in the USA report a large and record-breaking increase in visible light transmission efficiency of ultrathin materials called plasmonic metasurfaces, increasing their potential for use in a variety of optical applications. Plasmonic metasurfaces are patterned at scales below the wavelengths of light, supporting electric charge oscillations called plasmons. This can be used to manipulate light without using bulky optical elements for applications including atomic and molecular level sensing, optical computing and holography. Chunlei Guo and colleagues at the University of Rochester in New York designed, fabricated, and demonstrated the abilities of a metallic plasmonic metasurface with a light transmission efficiency over 40 percent - four times greater than has been achieved previously. Their advance overcomes limitations that have hindered attempts to exploit the fascinating properties of plasmonic metasurfaces.

## Introduction

Plasmonic metasurfaces (PMs) are two-dimensional arrays of metallic nanoantennas (meta-atoms) with subwavelength thicknesses and spacings and a spatially varying phase response^1,2^. PMs promise a paradigm shift in optics by replacing traditional bulky optical elements, e.g., beam deflectors^3–6^, lenses^7–10^, waveplates^11,12^, vortex generators^13–16^, and holograms^17–20^ with ultrathin, lightweight, and easy-to-integrate two-dimensional optical interfaces. Furthermore, PMs have an ultrathin thickness on the order of tens of nanometers, are easy to fabricate, support a field confinement beyond the diffraction limit, and have potential to respond on the timescale of a few femtoseconds^21^; these properties are unparalleled in traditional optical elements and cannot be easily extended to dielectric metasurfaces. Moreover, over the past two decades, the field of plasmonics has undergone significant advances and enabled a wide range of practical applications^21–23^. Applying the concept of PMs to these plasmonic devices and systems can further enhance their functionalities and performance, particularly in applications where plasmonics have unique advantages, such as superlens^24^, quantum plasmonics^25^, nonlinear optics^26^, photovoltaics^27^, nanolithography^28^, sensing^29^, life sciences, and medical applications^30^ (see the more detailed discussion on the advantages of PMs in the Supplementary Information). Among the demonstrated metasurfaces, geometric metasurfaces (GMs), consisting of anisotropic meta-atoms with identical geometric parameters and spatially varying orientations, provide an orientation-controlled and dispersion-less phase control over the entire 2*π* range for cross-circular polarization (CP) light^31^. GMs are favorable in practical applications as they are naturally broadband, easy to design, robust to fabrication errors and variations in material properties, and are able to control transmission and reflection^19,32–34^.

Although controlling optical transmission is of interest for many applications, the operation efficiency of ultrathin transmission-type GMs is severely limited. On the one hand, in an ultrathin antenna, only the electric dipole (ED) can be excited. Symmetric forward and backward scattering of the ED imposes a 25% theoretical upper limit on the cross-CP transmission, even when ignoring absorption losses^35,36^. Consequently, more than 75% of incident light is transmitted in the co-CP state, diffracted into undesired orders, or reflected. On the other hand, the notion that the metasurface efficiency can be increased by increasing the packing density of meta-atoms cannot be readily extended to GMs. At higher packing densities with smaller meta-atom interspaces, the near field of adjacent meta-atoms starts to couple and modifies the amplitude/phase responses of individual meta-atoms to deviate from their designed values. Accordingly, developing an optimal design of GMs at a high packing density is a formidable task. Furthermore, for plasmonic GMs, the efficiency is further limited by the strong absorption in metals, particularly in the optical band. Consequently, the demonstrated efficiencies for transmission-type plasmonic GMs in the visible and near-infrared regions are ≤10% for realizing various functions^37–40^. Realizing efficient PMs in the visible and near-infrared range, thus, is of paramount technological importance^41^.

Meta-atoms that simultaneously support ED and magnetic dipole (MD), so-called Huygens meta-atoms, are able to break the scattering symmetry and enhance the transmission efficiency^42,43^. In a Huygens meta-atom, zero backscattering is realized when the ED and MD are of the same magnitude and phase, as their back-scattered radiations destructively interfere, which satisfies the first Kerker condition^44^. However, plasmonic Huygens meta-atoms require metallic patterned multilayers, typically three layers or more^36,45–48^, which are difficult to fabricate, particularly when operating at short-wavelength optical range. In addition, previously reported Huygens meta-atoms are either isotropic or only capable of controlling linear polarization (LP) along a single direction. High-transmission GMs require the construction of a half-wave plate, in which the two orthogonal LP components of the incident CP light along the two main axes have a *π*-phase difference and maximum transmission^31^. The *π*-phase difference necessitates using anisotropic meta-atoms with nonoverlapping resonances at two orthogonal LPs, while maximum transmission requires satisfying the first Kerker condition at both LPs within the same wavelength range, which is difficult to achieve using anisotropic meta-atoms. The first Kerker condition can only be satisfied when the ED and MD are equal in magnitude and phase, which is a very restrictive condition, particularly for anisotropic meta-atoms. Consequently, increasing the transmission efficiency of GMs using the Huygens metasurface approach remains challenging. Other few-layer structures have been reported to have high transmission^49,50^. For example, by introducing two orthogonal gratings before and after a layer of metallic antennas, high-transmission PMs manipulating cross-LP light have been demonstrated in the terahertz^51^ and microwave^52^ regions. However, it is difficult to apply this three-layer scheme to GMs in the visible and near-infrared regions. Until now, there has been no direct solution to address the low transmission of plasmonic GMs in the visible and near-infrared regions. A reflection-type plasmonic GM reaching 80% efficiency in the visible and near-infrared regions has been reported by eliminating all the loss channels, except the inevitable absorption losses^19^. Ideally, a transmission-type plasmonic GM can reach an efficiency of 80% in the same wavelength region, if all undesired channels (i.e., reflection, co-CP transmission, and diffraction to undesired orders) are eliminated. Furthermore, absorption losses can be mitigated by designing the field distribution, as is shown in this work, or can be compensated by immersing the metasurface in a gain medium^53^.

**Fig. 1:**
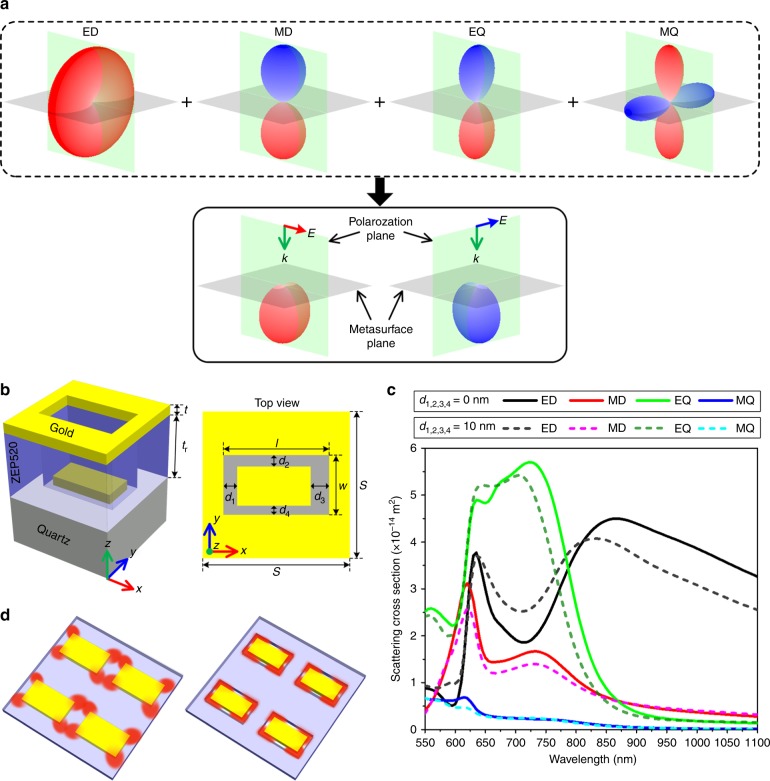
Concept and design of a multipole meta-atom for constructing a high-transmission GM. **a** Schematic showing radiation patterns of ED, MD, EQ, and MQ supported by a multipole meta-atom and their interference scattering for two orthogonal LPs at a specific wavelength in which a *π-*phase difference exists between the two LPs. Note that only the radiation for the transverse electric field component in the polarization plane is shown. The red and blue patterns represent out-of-phase fields. **b** Schematic showing the designed multipole meta-atom, which consists of a gold nanoaperture and a gold nanorod separated by a perforated ZEP520 layer. In the meta-atom, *S* = 320 nm, *l* = 230 nm, *w* = 130 nm, *t* = 35 nm, and *t*_r_ = 180 nm. The multipole response of this meta-atom can be tuned by introducing an air gap between the nanorod and ZEP520 sidewalls, i.e., a non-complementarity between the nanorod and the nanoaperture. The dimension of the air gap is denoted by *d*_1,2,3,4_ in four sides. **c** Calculated scattering cross sections contributed from each multipole for complementary (solid lines) and noncomplementary (dashed lines) MPMs with a RCP input. **d** Schematic showing the reduced near-field coupling between nanorods in the noncomplementary MPM

In this work, we use multipole meta-atoms that support not only ED and MD but also an electric quadrupole (EQ) and a magnetic quadrupole (MQ) to construct an ideal half-wave plate. As shown in Fig. 1a, in multipole meta-atoms, it is possible to completely suppress backscattering for both orthogonal polarizations by satisfying the so-called generalized Kerker condition^54^ and maintain the *π*-phase difference. This is the design principle of our work to be elaborated in this paper. We propose a multipole meta-atom design consisting of a metallic nanoaperture and a metallic nanorod separated by a perforated dielectric layer. The dimension of the nanorod (Fig. 1b) and thus the multipole response (Fig. 1c) can be modified by introducing a small air gap between the nanorod and the dielectric spacer separating adjacent meta-atoms. Tuning the meta-atom response allows us to simultaneously suppress backscattering for orthogonal LPs while maintaining an ~*π*-phase difference. Furthermore, we dramatically mitigate inter-meta-atom crosstalk and reduce metallic absorption due to confining the field within the introduced air gap, as shown in Fig. 1d (more detail will be given later). Based on the multipole meta-atom design, we demonstrate a transmission-type plasmonic GM with a theoretical efficiency of up to 45.5% at visible wavelengths. The ease of fabrication and high tolerance to fabrication errors of our multipole plasmonic metasurface (MPM) allowed us to experimentally achieve a transmission efficiency of 42.3% at 744 nm. We developed a beam deflector with an experimental transmission efficiency of 38.2% and extinction ratio (ER) of 7.8 dB over the co-CP transmission, which are ~400% and tenfold improvements, respectively, over those of the state-of-the-art plasmonic GMs in the visible and near-infrared regions (see Table 1). In addition, we demonstrate a hologram with a 37% transmission efficiency, which also represents a fourfold improvement over PM-based holograms^39^.

**Table 1:** Summary of previously reported transmissive geometric plasmonic metasurfaces in the visible and near-infrared regions. NS: not specified

Structure	Function	Wavelength (nm)	Efficiency	Extinction ratio	Reference
Nanorod	Deflector and vortex generator	670–1100	<10% according to ref. ^38^	−17 dB according to ref. ^16^	^5^
Nanorod	Metalens	740	5%	<0 dB (NS)	^7^
Nanorod	Hologram and metalens	880	5%	<0 dB (NS)	^10^
V-shaped	Vortex generator	780	3%	~ –10 dB	^13^
V-shaped	Vortex generator	780	8.6%	~ –10 dB	^14^
Split ring	Vortex generator	900	~5%	~ −10 dB	^15^
Nanoaperture	Vortex generator	632.8	3.3%	−4 dB	^16^
Nanorod	Hologram	810	4.5%	<0 dB (NS)	^20^
Nanorod	Polarization characterization	940	7.6%	<0 dB (NS)	^55^
Nanorod and nanoaperture	Deflector and hologram	744	38.2%	7.8 dB	This work

## Results

### Concept and design

In GMs, the transmission coefficient of cross-CP light is expressed as $t_{{\mathrm {cross - CP}}} = \frac{{t_x - t_y}}{2}{\mathrm {e}}^{ \pm i2a}$^31^, where + holds for a left circular polarization (LCP) input and – holds for a right circular polarization (RCP) input, *α* is the orientation angle of the meta-atom with respect to the *x*-axis, and *t*_*x*_ and *t*_*y*_ are the transmission coefficients for LP inputs along the *x*-axis and *y*-axis, respectively, when *α* = 0. Thus, to maximize the cross-CP transmittance (*T*_cross-CP_), which is proportional to |*t*_cross-CP_|^2^, the main goal is to realize phase(*t*_*x*_)−phase(*t*_*y*_)~*π* and high |*t*_*x,y*_|. For lossless or low-loss GMs, an ideal meta-atom is a half-wave plate with zero reflection at both LPs. After designing a meta-atom with high *T*_cross-CP_, an efficient GM also requires that the meta-atom supports phase responses solely controlled by its orientation and nearly identical amplitude responses for different orientations. To meet this condition, the coupling between adjacent meta-atoms should be negligible, as adjacent coupling can modify the meta-atom’s amplitude/phase response due to hybridization.

As mentioned earlier, based on the Huygens meta-atom approach, which depends on the destructive interference of the back-scattered light from the ED and MD^44,56^, it is difficult to simultaneously satisfy the *π*-phase-difference and zero-reflection conditions. To overcome this problem, we use meta-atoms that support multipole moments. As shown in Fig. 1a, zero reflection for both LPs is possible if the multipole moments destructively interfere in the backward direction, which leads to the definition of a generalized Kerker condition^54,57^. Figure 1b shows the design of a multipole meta-atom, which is composed of a rectangle-shaped gold nanoaperture and a gold nanorod separated by a perforated dielectric resist layer with an etched hole. The nanorod is situated in the etched hole. The nanoaperture has the same dimension as the etched hole, while the dimension of the nanorod can be tuned to be smaller, i.e., non-complementarity between the nanorod and nanoaperture can be introduced by introducing an air gap between the nanorod and the sidewall of the etched hole. The dimensions of the air gap are represented by the gap sizes on the four sides, which are defined as *d*_1_, *d*_2_, *d*_3_, and *d*_4_. The separation between the two gold layers is *t*_r_. *S* is the subperiod of the meta-atom. *l* and *w* are the length and width of the nanoaperture, respectively. *t* is the gold thickness. Unless otherwise stated, *S* = 320 nm, *l* = 230 nm, *w* = 130 nm, *t* = 35 nm, and *t*_r_ = 180 nm in all calculations.

To uncover the importance of tuning the multipole excitations with the air gap, i.e., non-complementarity, we study a complementary meta-atom design without an air gap, i.e., *d*_1,2,3,4_ = 0 nm, and a noncomplementary design with a simple symmetric air gap of *d*_1,2,3,4_ = 10 nm. By using an exact multipole expansion approach^58^, we can calculate the multipole response of these meta-atoms. Figure 1c shows the calculated scattering cross sections of the ED, MD, EQ, and MQ in complementary (solid lines) and non-complementarity (dashed lines) meta-atoms, which indicates that both meta-atoms sustain multipole resonances in the visible and near-infrared regions. In particular, the high-order EQ is comparable or even stronger than the ED and MD; therefore, it contributes to the multipole interference. More importantly, the multipole responses, and correspondingly, their interference are tunable by introducing non-complementarity. As shown later, this tunability can facilitate destructive interference in the backward direction, i.e., satisfying the generalized Kerker condition.

Another advantage of the noncomplimentary meta-atom is that by introducing non-complementarity the near field of the nanorod is strongly confined within the air gap instead of the dielectric spacer and metallic nanorod (Fig. 1d). This result is a consequence of the continuity of the displacement field normal component, which results in a higher-field intensity for regions with lower electric permittivity, e.g., air, and is analogous to hybrid plasmonic structures^59^. The reduced field inside the resist spacer minimizes the near-field coupling between nanorods in adjacent meta-atoms, which allows an increase of the meta-atom packing density and hence the metasurface efficiency. Furthermore, the reduced field inside the nanorod decreases metallic absorption, which further increases the efficiency.

For GMs, we are concerned with the satisfaction of the generalized Kerker condition for two orthogonal LPs and the phase difference between them. We define the generalized Kerker coefficients as^57^

1 $$K_x = \left( {1 + r_{as}{\mathrm {e}}^{i2kz_0}} \right)\left( {p_x - \frac{{ik}}{{2c}}M_{yz}} \right) - \left( {1 - r_{as}{\mathrm {e}}^{i2kz_0}} \right)\left( {\frac{{m_y}}{c} - \frac{{ik}}{6}Q_{xz}} \right)$$

for the *x*-LP input along the –*z* direction and

2 $$K_y = \left( {1 + r_{as}{\mathrm {e}}^{i2kz_0}} \right)\left( {p_y + \frac{{ik}}{{2c}}M_{xz}} \right) - \left( {1 - r_{as}{\mathrm {e}}^{i2kz_0}} \right)\left( {\frac{{m_x}}{c} + \frac{{ik}}{6}Q_{yz}} \right)$$

for the *y*-LP input along the –*z* direction, where *r*_as_ = (1–*n*_s_)/(1 + *n*_s_) is the reflection coefficient from the air cladding to a substrate with a refractive index *n*_s_, *z*_0_ is the distance between the multipole expansion center and the substrate interface, and *k* and *c* are the wavenumber and light speed in air, respectively. *p*, *m*, *Q*, and *M* are the ED, MD, EQ, and MQ moments, respectively. The back-scattered far field from a meta-atom is proportional to *K*_*x*,*y*_. Accordingly, the generalized Kerker condition for zero reflection is fulfilled when *K*_*x*,*y*_ = 0^57^.

**Fig. 2:**
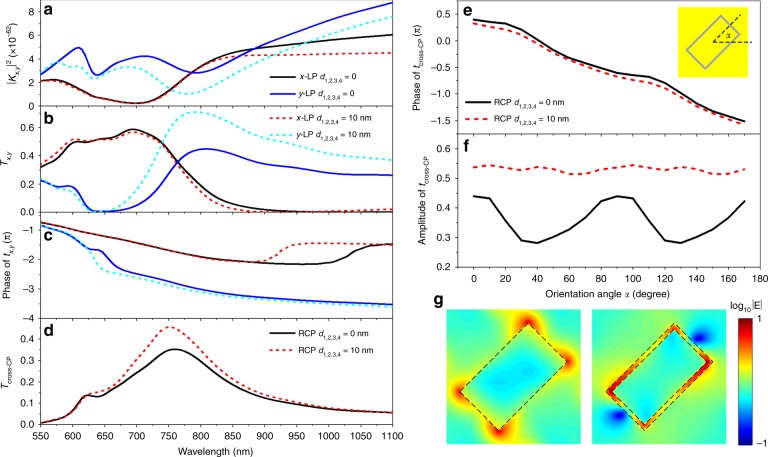
MPM approaching the generalized Kerker condition and reducing adjacent coupling. **a–d** Simulated generalized Kerker coefficients (**a**), transmittance (**b**), and phase of transmission coefficients (**c**) with LP inputs, and cross-CP transmittance (**d**) with a RCP input for a complementary MPM with *d*_1,2,3,4_ = 0 (solid lines) and a noncomplementary MPM with *d*_1,2,3,4_ = 10 nm (dashed lines). Both designs support a *T*_cross-CP_ over 25% over a broad wavelength range near 750 nm due to the high *T*_*x*,*y*_ and phase difference near *π* between *t*_*x*_ and *t*_*y*_. By introducing non-complementarity, *T*_cross-CP_ is increased by over 10% due to approaching the generalized Kerker condition for *y*-LP, thus increasing *T*_*y*_ and ensuring large *T*_*x*_ and *T*_*y*_ values within the same wavelength range. **e**, **f** Simulated phase (**e**) and amplitude (**f**) of the cross-CP transmission coefficients with a RCP input for meta-atoms with different orientations. By introducing non-complementarity, the amplitude response for different orientations becomes relatively constant due to the reduced adjacent coupling between nanorods. **g** Electric field distributions on the nanorod layer for complementary and noncomplementary meta-atoms with *α* = 45° and a RCP input. In all simulations, the input is along the –*z* direction

Figures 2a–c show the calculated generalized Kerker coefficients (*K*_*x*,*y*_), transmittances (*T*_*x*,*y*_), and phase of *t*_*x*,*y*_, respectively, at two LPs for complementary and noncomplementary meta-atoms. The calculations of *p*, *m*, *Q*, and *M* at two LPs are given in the Supplementary Information (SI) Fig. S1. Clearly, the local minima of *K*_*x*,*y*_, i.e., where the meta-atom approaches the generalized Kerker condition, correspond to the transmission peaks for *T*_*x*,*y*_ in all cases. For the complementary design, *K*_*x*_ is minimum at *λ*~ 700 nm and *K*_*y*_ is minimum at *λ*~800 nm, corresponding to transmission peaks for *T*_*x*_ of ~59% and *T*_*y*_ of ~45%, respectively. Between the two transmission peaks, the metasurface has a high *T*_*x*_ and *T*_*y*_. At the same time, the phase difference between *t*_*x*_ and *t*_*y*_ is ~*π* in the same wavelength range, which makes the design behave as an efficient half-wave plate in the transmission direction, with a maximum *T*_cross-CP_ of 35.2% at *λ*~760 nm (Fig. 2d), i.e., with an efficiency 10% higher than the 25% limit for ultrathin metasurfaces. The calculation described in SI Fig. S2 shows that the meta-atom works as an efficient half-wave plate in the reflection direction as well at the same wavelength, with a cross-CP reflection efficiency of 37.7%. In both directions, the unwanted co-CP lights are <1%. Accordingly, the designed complementary MPM can realize a combined function of a half-wave plate and a 50:50 beam splitter with a net cross-CP conversion efficiency of 73%, which is close to that of the state-of-the-art PMs operating in the reflection mode^19^ and dielectric metasurfaces operating in the transmission mode^32–34^.

However, in most applications, wavefront control of the transmitted light with high efficiency is preferred. In our design, the introduction of non-complementarity between the nanoaperture and nanorod is the key to engineering the multipole interference and enhancing the *T*_cross-CP_. As shown in Figs. 2a, b and Fig. S1, the effect of introducing non-complementarity on the multipole excitations of the *x*-LP input is almost negligible in the wavelength range of interest. However, for the *y*-LP input, the non-complementarity results in a reduced *K*_*y*_ and enhanced *T*_*y*_ within the wavelength range of interest, i.e., the wavelength range where *T*_*x*_ is maximum. The corresponding *y*-LP transmission peak is increased to *T*_*y*_ ~71% at 780 nm, which is closer to the peak wavelength of *T*_x_ at 705 nm (see the multipole radiations and their interferences at 705 nm for *x*-LP and 780 nm for *y*-LP in SI Fig. S3). Meanwhile, a phase difference of ~*π* is still maintained (Fig. 2c). As a result, we achieve an overall enhancement in the *T*_cross-CP_ of the noncomplementary design (Fig. 2d). The peak efficiency (*E*_p_) is increased to 45.5% at a peak wavelength (*λ*_p_) of 751 nm, i.e., *T*_cross-CP_ is increased by >10%. Here, we note that the 26% *T*_*y*_ increase is not only due to approaching the generalized Kerker condition, which decreases the reflection by 14%, but also due to a reduction in absorption at the transmission maximum of ~12% (SI Fig. S4).

The reduced near-field coupling between the meta-atoms after introducing non-complementarity is evident in Figs. 2e, f, which shows the calculated phase and amplitude (*λ* = 750 nm) of *t*_cross-CP_, respectively, as a function of the orientation angle (*α*) for complementary and noncomplementary meta-atoms (see the broadband calculation in SI Fig. S5). Both designs support good orientation-controlled phase responses. However, the *t*_cross-CP_ amplitude of the complementary design significantly changes as a function of *α*, reaching a minimum at an *α* of ~40°, where the meta-atom interspace is the minimum (SI Fig. S6), i.e., the near-field coupling is the maximum. On the contrary, the noncomplementary meta-atom has higher and relatively flat amplitudes. Figure 2g shows the electric field distributions of the nanorod layer for complementary (left) and noncomplementary (right) meta-atoms with *α* = 45°. The localized field inside the air gap mitigates the crosstalk between noncomplementary meta-atoms. The reduced coupling of noncomplementary meta-atoms allows further increases of the efficiency by using a higher packing density, while for the complementary design, the efficiency decreases (SI Fig. S7). Note that meta-atom crosstalk does not always degrade the performance of metasurfaces that do not adopt the GM scheme. For example, strong coupling has been deliberately designed to improve the cross-LP transmission in a V-shaped bilayer metasurface^60^. The adopted meta-atoms with a combination of solid and inverse structures share some of the aspects of our complementary design, but our work is mostly focused on a noncomplementary design. Furthermore, a strong-coupling design will always impose complex designs and stringent requirements on the fabrication accuracy and cannot be used for GMs.

### Fabrication and tolerance

**Fig. 3:**
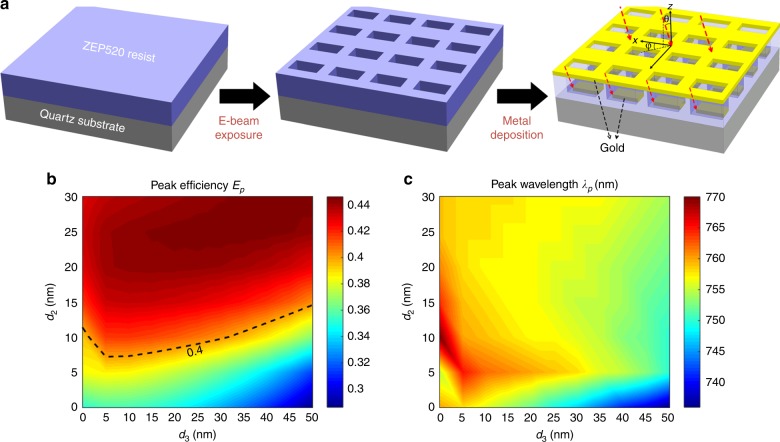
Fabrication method and high fabrication tolerance. **a** Fabrication procedure of the noncomplementary MPM with *d*_1,4_ = 0 and *d*_2,3_ = 20 nm, in which a tilt metal deposition technique is utilized. The red dashed arrows indicate the metal deposition direction. In this case, the nanorod dimension mismatch values are expressed by *d*_1,4_ = 0, *d*_2_ = *t*_r_ tan*θ* sin*φ*, and *d*_3_ = *t*_r_ tan*θ* cos*φ*. **b**, **c** Simulated transmission efficiency maximum *E*_p_ (**b**) and related wavelength *λ*_p_ (**c**) of the noncomplementary MPM as functions of *d*_2_ and *d*_3_ when *d*_1,4_ = 0. The black dashed line in (**b**) marks the contour of 0.4. *E*_p_ is higher than 40% at visible wavelengths for a wide range of mismatch values

The noncomplementary design can be realized with the facile fabrication method described in Fig. 3a. First, rectangle-shaped patterns were defined on the ZEP520 resist layer by electron beam lithography (EBL). Then, a thin gold layer was deposited by electron beam evaporation. The noncomplementary nanorod was realized by tilting the stage by a small angle and halting the stage rotation. To realize *d*_2,3_ = 20 nm for the MPM with *α* = 0°, a small tilt with an azimuthal angle *φ* = 45° and a polar angle *θ* ≈ 9° was applied. To avoid gold deposition on the resist sidewalls, a dimension bias and a high dose were applied during the EBL to achieve a slight undercut profile of the resist sidewalls^61^. This fabrication procedure is simpler than that for single-layer PMs, which requires an additional step to remove unwanted metals by a lift-off or etch-back process^62^ (see the section “Materials and methods” for more details). Note that when adopting the tilt deposition technique shown in Fig. 3a, *d*_1_ and *d*_4_ will be zero. However, even when *d*_1,4_ = 0, one can see from Fig. 3b that *E*_p_ maintains high values (>40%) for a wide range of values of *d*_2_ and *d*_3_. Meanwhile, *λ*_p_ remains within the wavelength range of interest, as shown in Fig. 3c. Figures 3b–c highlight the high tolerance of our design to fabrication errors. The performance of the noncomplementary design shows high tolerance to other parameters as well (SI Figs. S8 and S9). Because a large deposition tilt may cause metal deposition on the sidewalls and an observable chirality, we use a relatively small air gap with *d*_1,4_ = 0 and *d*_2,3_ = 20 nm. Such an asymmetric noncomplementary design supports the multipole response as well and a theoretical *E*_p_ of 44.3% at 757 nm (SI Fig. S10), which provides a similar efficiency improvement to the symmetric case with *d*_1,2,3,4_ = 10 nm. The complementary design is obtained by enabling stage rotation during deposition.

### Demonstration of efficient cross-CP transmission

**Fig. 4:**
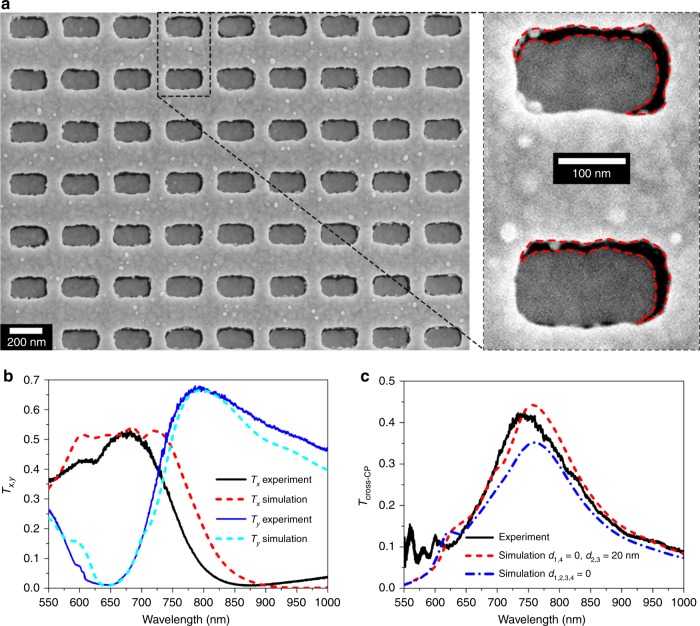
Experimental demonstration of a noncomplementary MPM with high *T*_cross-CP_. **a** SEM image of a fabricated MPM and an enlarged view of two meta-atoms, in which asymmetric air gaps are clearly observable. The red dashed lines mark the edges of the air gaps. **b**, **c** Simulated and measured LP (**b**) and cross-CP (**c**) transmittances. The solid, dashed, and dash-dotted lines represent the experimental results, simulation of the noncomplementary MPM with *d*_1,4_ = 0 and *d*_2,3_ = 20 nm, and simulation of the complementary MPM, respectively. The measured efficiency is higher than that for the simulation of the complementary MPM by ~7%, confirming the role of tuning the multipole response in approaching the generalized Kerker condition

Figure 4a shows scanning electron microscopy (SEM) images of the fabricated noncomplementary MPM with *d*_1,4_ = 0 and *d*_2,3_ = 20 nm. The measured and calculated *T*_*x*,*y*_ are shown in Fig. 4b, and they are in good agreement. *T*_*y*_ is experimentally found to be ~68% at 790 nm, which indicates the efficient suppression of back-scattered *y*-LP light. The details of the measurement setup are provided in the “Materials and methods” and SI Fig. S19. Figure 4c shows the *T*_cross-CP_ with the RCP input. The black solid, red dashed, and blue dash-dotted lines represent the experimental result, simulation of noncomplementary MPM, and simulation of complementary MPM, respectively. The demonstrated *T*_cross-CP_ exceeds 10% over a wide wavelength range of 630–970 nm. In particular, *E*_p_ is 42.3% at 744 nm. The measured *E*_p_ is larger than the simulation value of the complementary design (35.2% at 760 nm) by ~7% but lower than the simulation value of the noncomplementary design (44.3% at 757 nm) by ~2%. This small deviation is likely due to the chromium adhesion layer and a slightly tilted edge surface of the metal layer, which are omitted in the simulation. Similar results of *T*_cross-CP_ for the LCP input are obtained (SI Fig. S11). The noise in the measured *T*_cross-CP_ below 630 nm is due to the bandwidth limitation of the quarter-wave plate used in the experiment (see the section “Materials and methods”).

### Application in beam deflection

**Fig. 5:**
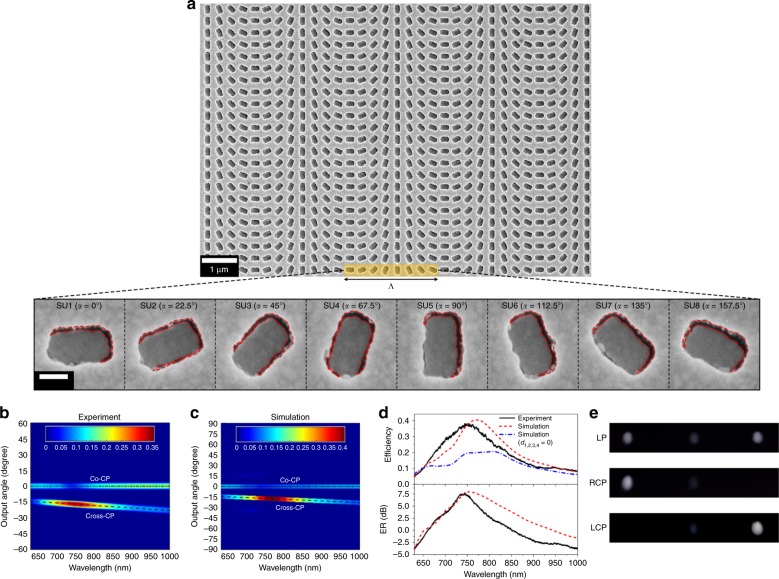
Experimental demonstration of an efficient beam deflector with reduced adjacent coupling. **a** SEM images of the fabricated GM and an enlarged view of one unit cell. Each SU with a different orientation shows different values of *d*_1,2,3,4_. The red dashed lines mark the edges of the air gaps. **b**, **c** Measured (**b**) and simulated (**c**) transmittances of cross-CP and co-CP light as functions of the wavelength and output angle for a RCP input with normal incidence. The dashed lines represent the theoretical diffraction angle of the anomalous diffractions. **d** Measured and simulated deflection efficiencies of anomalous diffraction in transmission and the related ER over normal diffraction. The solid, dashed, and dash-dotted lines represent the experimental results, simulation of noncomplementary GM, and simulation of complementary GM, respectively. In the simulation of the noncomplementary GM, the *d*_1,2,3,4_ values of each SU are set as the averaged values measured in (**a**). Detailed values are provided in SI Fig. S12b. The measured efficiency is higher than that from the simulation of the complementary GM by ~17.5%, which is larger than the improvement of the regular MPM described in Fig. 4 and confirms the benefit of the noncomplementary design in reducing adjacent coupling. **e** Far-field diffraction patterns of the beam deflector at 750 nm for LP, RCP, and LCP inputs

By introducing a phase gradient on the surface, we test our MPM as a beam deflector. Figure 5a shows a SEM image of the fabricated GM and an enlarged view of one unit cell. A single unit cell consists of eight subunits (SUs) with an orientation step of 22.5° (period: Λ = 2.56 μm). Note, the dimension mismatches (i.e., *d*_1,2,3,4_) for SUs with different orientations differ during the tilted deposition. However, the high efficiency persists for each SU because it is relatively insensitive to the values of *d*_1,2,3,4_, as we showed earlier (Figs. 3b, c and Fig. S8). The average mismatch values of each SU measured from the SEM image shown in Fig. 5a are used for the simulations. The detailed simulation structure is described in SI Fig. S12b. Figure 5b shows the experimentally measured *T*_cross-CP_ and *T*_co-CP_ as functions of the wavelength and output angle for the RCP input with a normal incidence, which agree with the corresponding simulation result presented in Fig. 5c. The black dashed lines represent the theoretical output angle of the anomalous beam, which is determined by arcsin(*λ*/Λ)^27^. Figure 5d shows the measured beam deflection efficiency of the anomalous transmission and ER, defined as 10 × log_10_(*T*_cross-CP_/*T*_co-CP_), and the corresponding theoretical calculations for the noncomplementary and complementary designs. The theoretical (40.4%) and experimental (38.2%) *E*_p_ of the noncomplementary design are higher than the theoretical (20.7%) *E*_p_ of the complementary design. In addition, the measured efficiency exceeds the 25% limit for ultrathin metasurfaces over the wide 690–810-nm range. The measured ER peaks at 7.8 dB at ~745 nm and exceeds 0 dB between 660 and 850 nm. Both *E*_p_ and ER are significantly higher than the values of the current state-of-the-art plasmonic GMs (*E*_p_ ≤ 10%, ER **≤** 0 dB) in the visible and near-infrared regions in realizing various functionalities^5,13,15,55^. Furthermore, the close agreement between the theoretical simulation (*E*_p_ = 40.4%, ER = 8 dB) and experimental measurement (*E*_p_ = 38.2%, ER = 7.8 dB) indicates the tolerance of our design to fabrication errors. The beam deflection results for the LCP input are very similar (SI Fig. S13).

We note that the experimental *E*_p_ of the noncomplementary MPM was ~7% larger than the theoretical *E*_p_ of the complementary design shown in Fig. 4c due to approaching the generalized Kerker condition. However, the experimental *E*_p_ of the noncomplementary GM is ~17.5% larger than the theoretical *E*_p_ of the complementary GM design. This additional enhancement in the transmission efficiency benefits from reducing the near-field coupling of the meta-atoms. In GMs, nonuniform amplitude responses resulting from coupling-induced modifications can decrease the total transmittance and increase the portion of the transmitted power into undesired diffraction orders. The reduced coupling between meta-atoms is shown by the relatively flat-amplitude responses of eight SUs and the electric field distributions in one unit cell of both complementary and noncomplementary GMs at *λ* = 750 nm in Fig. S12. The simulations described in SI Figs. S14 and S15 show that diffractions to undesired high orders for the noncomplementary GM are less than one-tenth of the zero order for both cross-CP and co-CP. In the experiment, the high-order diffractions are within the noise level of the spectrometer.

Figure 5e shows the far-field diffraction patterns at 750 nm with the LP, RCP, and LCP inputs (see SI Fig. S16 for the results at various wavelengths). The central spots and the spots on each side represent co-CP normal diffraction and cross-CP anomalous diffraction beams, respectively. In contrast to previous experiments using single-layer PMs^3–5,15,55,63^, the anomalous diffracted beams from the GM deflector are stronger than the normal beams, which is consistent with the broadband experiments described in Figs. 5b and S13b.

### Application in a hologram

**Fig. 6:**
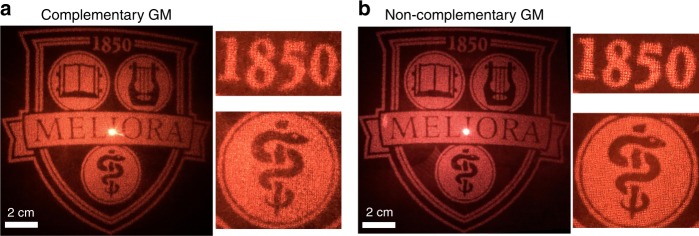
Experimental demonstration of a hologram. **a**, **b** Holography images at 750 nm for the complementary GM (**a**) and noncomplementary GM (**b**). The enlarged views clearly show the better quality of the holography images obtained with the noncomplementary GM

We demonstrate the versatility of the proposed design by realizing a record-high efficiency hologram using a plasmonic GM. The phase pattern of the target image, the logo of the University of Rochester, is computed by means of the Gerchberg−Saxton phase-retrieval algorithm^64^. Figure 6 shows experimental holography images in the far field at *λ* = 750 nm. Figures 6a and 6b are obtained from complementary and noncomplementary plasmonic GMs, respectively. While both designs show a clear reconstruction of the target image, the noncomplementary design offers a higher transmission efficiency and clearer details when looking at the enlarged view. This improvement is due to the aforementioned efficiency enhancement and smaller amplitude modification from the reduced adjacent coupling. The hologram efficiencies are measured to be 26.7% and 37% for the complementary and noncomplementary designs, respectively. These results are also better than those of the state-of-the-art GMs in the visible and near-infrared regions^39^. The hologram is broadband as well, as we demonstrate in SI Fig. S17, which shows holography images at various wavelengths. The bright spots in the center of the reconstructed images arise from the zero-order light not coupling into the metasurface due to beam overfilling.

## Discussion

A significant increase in the transmission efficiency of plasmonic GMs is facilitated by tuning the multipole response of individual meta-atoms and by minimizing the crosstalk between meta-atoms. The maximum efficiency of the MPM is on the long-wavelength side of the visible spectrum, 744 nm. However, the demonstrated high efficiency exceeds that of the state-of-the-art GMs over a wide wavelength range of 630–970 nm, i.e., in the red-color range of the visible (visible covers 380–780 nm^65^) and near-infrared regions. This concept and these techniques can be used for shorter visible wavelengths (see a design in SI Fig. S18) or longer-wavelength regions by tuning the dimensions of the meta-atom. This concept can be used for dielectric metasurfaces as well. The generalized Kerker condition, in principle, allows for perfect transmission, reflection, or absorption^54^. Accordingly, the transmission efficiency can be increased further. By eliminating all co-CP transmissions and co-CP and cross-CP reflections with further optimization, it is possible to enhance the transmission efficiency to >70% and ensure that it is only limited by the intrinsic absorption losses in metals. Absorption can be mitigated by storing the field inside an introduced air gap, as we demonstrated, or by using metals with lower Ohmic losses. For PMs operating at longer wavelengths beyond the IR range with low absorption losses, it is possible to achieve almost 100% transmission with a MPM, which is simpler to fabricate compared with transmit arrays and multilayer metasurfaces. Furthermore, reflective-type metasurfaces can benefit from the generalized Kerker, no-front-scattering condition. On the other hand, perfect and selective light absorption can be achieved by eliminating both reflection and transmission, using the generalized Kerker approach. In the future, even higher-order multipoles can also be included to improve the performance of PMs^66,67^.

## Materials and methods

### Fabrication

Fabrication was carried out at the Cornell Nanoscale Facility. The metasurface was fabricated on a 500-μm-thick quartz wafer from Mark Optics at Santa Ana, CA, USA. After cleaning with acetone and isopropyl alcohol, a layer of positive electron beam resist ZEP520 with a thickness of 180 nm was spin-coated on the substrate, followed by baking for 2 min at 170° on a hot plate. Then, a thin layer of conductive polymer Espacer was spin-coated to dissipate charge in the EBL process. Next, rectangle-shaped nanostructure patterns were defined on the resist layer by EBL on a JEOL 9500 system (100 kV, 1 nA). The patterned area was 400 × 400 μm for the deflector and 655 × 655 μm for the hologram. A dimension bias (25 nm) and a relatively high dose (380 μC/cm^2^) were applied to achieve a slight undercut profile of the resist. After EBL, the Espacer was removed by water rinsing. Low-temperature development with high contrast was implemented by successively developing the sample in ZED-N50 for 45 s, in methyl isobutyl ketone for 30 s, and in isopropyl alcohol for 30 s. The sample was then dried by a nitrogen blow. After development, 3 nm of chromium and 32 nm of gold were deposited in an electron beam evaporation system (Kurt J. Lesker PVD 75) that had a stage that could be tilted and rotated. In the tilt deposition, there was no metal deposition on the bottom layer in the shadow area of the resist. Theoretically, for a deposition direction along −(sin *θ* cos *φ*, sin *θ* sin *φ*, cos *θ*) determined by the azimuthal (*φ*) and polar (*θ*) angles, the shadow dimensions (i.e., mismatch values) can be expressed by *t*_r_ tan *θ|*sin(*φ−α*)| and *t*_r_ tan *θ|*cos(*φ−α*)| in two directions for a meta-atom with an orientation angle *α*. In the fabrication of noncomplementary MPM and GM, the sample rotation was disabled, and a small sample tilt of *φ* = 45° and *θ* ≈ 9° was applied during deposition. In the fabrication of the complementary GM for the hologram, stage rotation was enabled. No liftoff was processed after deposition. The fabricated metasurface was characterized by a scanning electron microscope (Hitachi S-4100). All the SEM images were taken at normal incidence to the sample surface.

### Simulation

Simulations were performed using the commercially available finite-difference time-domain software from Lumerical. In the simulations, LP or CP light was injected from the top in air. The CP input source and spin state were realized by adding two linear polarized sources in the *x* and *y* directions and setting the initial phase between them to ±*π*/2. The periodic and perfectly matched-layer boundary conditions were applied along the transverse and longitudinal directions with respect to the propagation of light. The dielectric constants of the gold and quartz substrates were directly obtained by using the Gold–Johnson and Christy database and the SiO_2_-Palik database in the software material's library, respectively. The refractive index of ZEP520 resist was obtained from spectroscopic ellipsometry measurements.

### Measurement

The measurement setups are shown in SI Fig. S19. In the broadband measurements, a collimated beam from a broadband lamp source (Thorlabs: SLS301, wavelength range: 360–2700 nm) was focused onto the sample, which was mounted on a three-dimensional moveable and rotatable stage. Before the focusing lens, a broadband Glan–Thompson polarizer (Melles Griot: 03 PTH 112/C, wavelength range: 350–2300 nm) and a quarter-wave plate (Thorlabs: AQWP10M-980, wavelength range: 630–1200 nm) were utilized to generate CP. The transmitted beam was collected by an objective (×10, 0.25). The spin state of the output was selected by another broadband quarter-wave plate and polarizer set. The quarter-wave plates were only inserted into the setup during the CP measurements. The beam was finally focused into the fiber collector of a spectrometer (Photon Control: SPM001, wavelength range: 300–1000 nm). An iris was used to select the part of the light that passed through the metasurface area. Therefore, this setup was able to measure the LP and CP transmissions of the metasurface over wavelength ranges of 360–1000 and 630–1000 nm, respectively. The transmittance of the metasurface was normalized to the transmittance through the quartz substrate only, which was ~93% in the studied wavelength range.

In the CW deflection and hologram experiments, a CW output at 730–800 nm was obtained from a Griffin Ti–Sapphire femtosecond laser (KM-Labs, Inc.) by setting the laser to work in the CW mode. The wavelength was tunable from 730 to 860 nm. The CW at 632.8 nm was from a collimated helium–neon laser. In the deflection experiment, the CW beams were focused on the sample and the output diffraction beams were recorded by a CCD camera (General Electric: SITE GARD II) in the far field. In the hologram experiment, the CW beams were shrunk by a 4-f system before being shone onto the metasurface and the holography images were recorded by a cell phone camera on a whitepaper screen located 11.5 cm after the sample. The efficiency was measured by a power meter.

## Supplementary information

Supplementary Information_LSA20190292R
