# Supplementary material for: Plasmonic metasurfaces with 42.3% transmission efficiency in the visible
Source: Light Sci Appl. 2019 Jun 12;8:53. doi: 10.1038/s41377-019-0164-8 (PMC6559953; doi:10.1038/s41377-019-0164-8)
Supplement: Supplementary file 1 — Supplementary Information_LSA20190292R [file 41377_2019_164_MOESM1_ESM.docx]

**Supplementary Information for**

**Plasmonic metasurfaces with 42.3% transmission efficiency in the visible**

Jihua Zhang^1^, Mohamed Elkabbash^1^, Ran Wei^1^, Subhash C. Singh^1,2^, Billy Lam^1^, and Chunlei Guo^1,2,*^

^1^The Institute of Optics, University of Rochester, Rochester, New York, 14627, USA

^2^Changchun Institute of Optics, Fine Mechanics, and Physics, Chinese Academy of Sciences, Changchun 130033 China

*Corresponding email: guo@optics.rochester.edu

## Advantages of plasmonic metasurfaces in specific applications

Although dielectric metasurfaces have become a trend in wavefront shaping due to their low loss and high efficiency^1^, plasmonic metasurfaces (PMs) still have unique advantages in specific wavelength ranges and applications. For example, PMs can work in microwave range with nearly 100% efficiency^2^. In addition, the thickness of the constituent metallic structure can be down to tens of nanometers, while the dielectric meta-atoms typically have thickness on the order of hundreds of nanometers. The ultrathin feature makes their fabrication easy. The most important advantages come from their ability to support strong field confinement in a scale beyond the diffraction limit^3^ and their potential to respond on the timescale of a few femtoseconds^4^, which make them preferred in specific applications such as merging electronics and photonics at the nanoscale^5^, sensing^6^, nonlinear^7^, ultrafast^4^, nanolithography^8^, and spontaneous emission modification^9^.

For sensors, the most important figure-of-merit is the sensitivity. Typically, nanoantennas are used to detect the change of external environment in their near-fields. For dielectric nanoantennas, the field maximum is located in the antenna center, i.e. inside the antenna. While for plasmonic nanoantennas, the field maximum is located at its surface, i.e. outside of the antenna. This field distribution makes sensing the electromagnetic environment an intrinsic property of plasmonic structures. Plasmonic nanoantennas are intrinsically more sensitive than dielectric ones. Therefore, while biosensing has been realized using dielectric nanoresonators, plasmonic-structures based biosensors offer far better sensitivity^10,11,12^.

Plasmonic nanoantennas are intrinsically favorable for high-efficiency nonlinear applications due to their strong local field enhancement in subwavelength scale and high sensitivity to the refractive indices of metals and surrounding materials^7^. Dielectric materials with low loss may have stronger nonlinear effects in a long-range bulk material or waveguide. At the subwavelength scale, a metasurface comprised of plasmonic nanoantennas can support higher nonlinear efficiency than the dielectric ones. The most efficient nonlinear metasurfaces are based on the PMs^13,14,15^.

For ultrafast application, the response time is a key property. Plasmonic excitations can respond on the timescale of a few femtoseconds^4^. For example, a sub-100-fs plasmonic switching has been reported in gold nanoantennas^16^. Sub-picosecond plasmonic switching has also been demonstrated in indium tin oxide nanorod arrays^17^. In switchable dielectric metasurfaces based on semiconductor, phase change material, or mechanical tuning, the response time is typically longer than picosecond level^18^. Although sub-picosecond response was obtained using dielectric metasurfaces based on two photon absorption^19^, the versatility and inherent ultrafast response of plasmonic nanoantennas over wide range of wavelengths makes them a favorable platform for ultrafast photonics.

In addition, plasmonic structures have been successfully applied to nanolithography^8^. In this application, plasmonic structures are preferred as their ability to realize patterns beyond the diffraction limit. Furthermore, plasmonic nanoantennas can be employed to control the spontaneous emission of quantum emitters^9^. For this application, plasmonic nanoantennas consistently provide significantly higher Purcell enhancements due to their ability to concentrate electromagnetic energy to a small volume^20^.

Due to these unique advantages in specific applications, improving the efficiency of PMs and making it to the same level of dielectric metasurfaces is of paramount importance to reinforce the technological relevance of these plasmonic applications.

## Multipole excitations of complementary and non-complementary MPMs


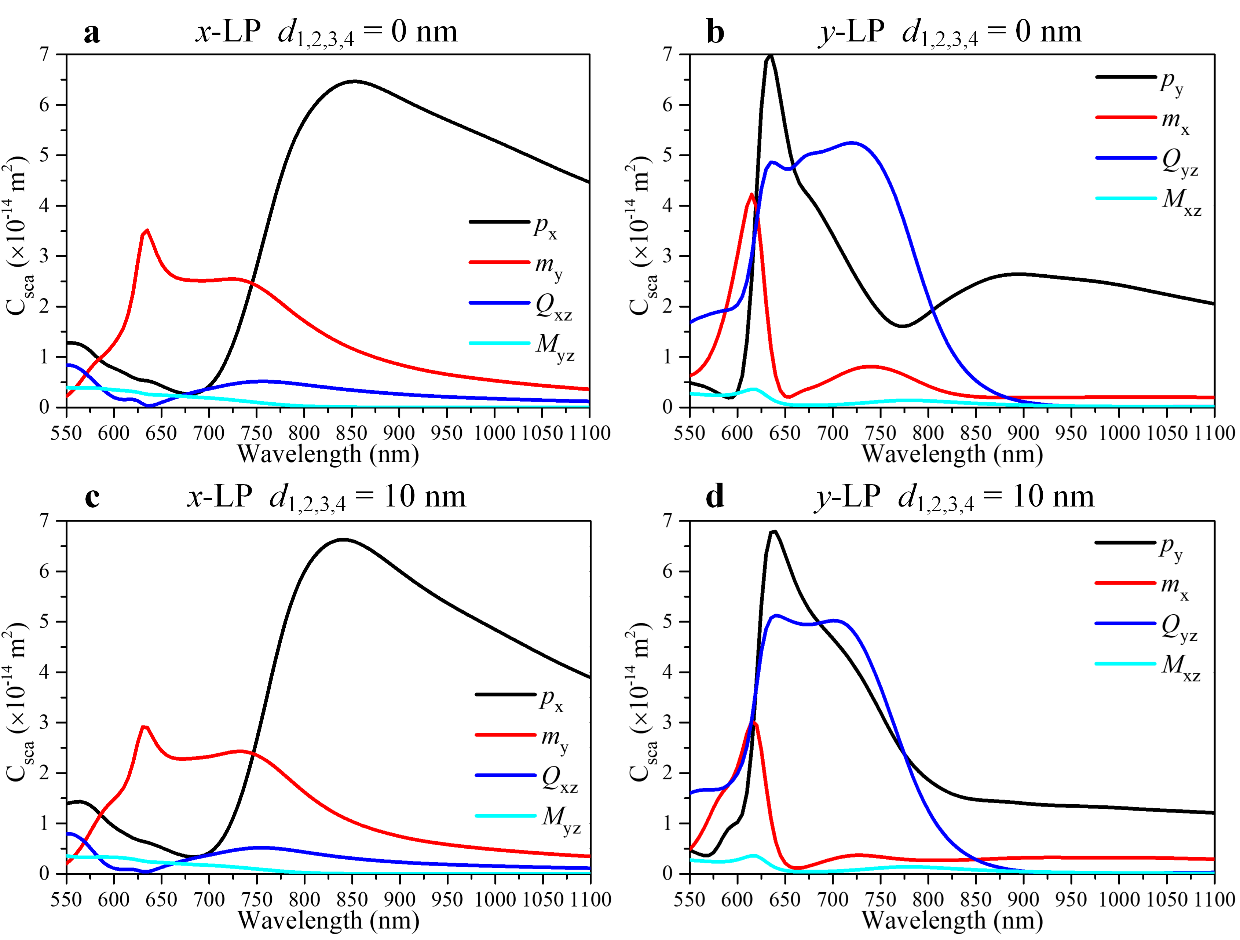


**Figure S1.** Calculated scattering cross sections (*C*_sca_) contributed from each multipole moment with *x*-LP (**a**, **c**) and *y*-LP (**b**, **d**) inputs, for the complementary (*d*_1,2,3,4_ = 0 nm) and non-complementary (*d*_1,2,3,4_ = 10 nm) MPMs.

## Efficient net cross-CP conversion of the complementary MPM


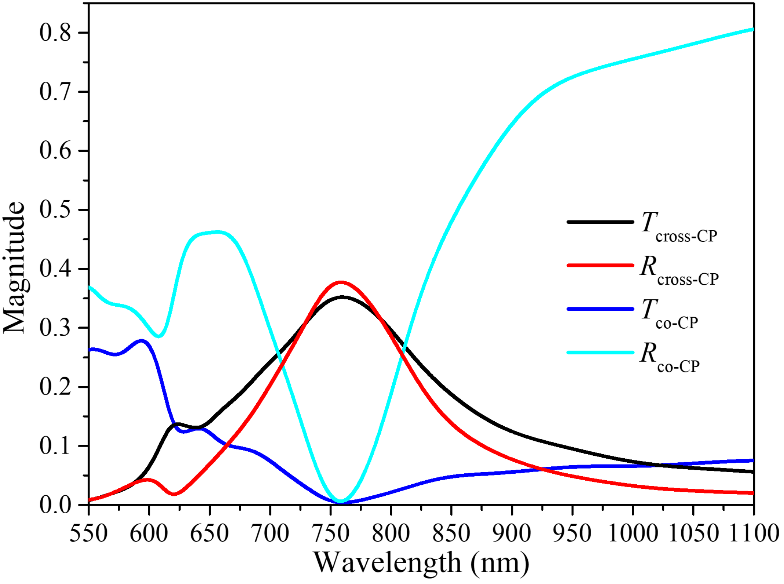


**Figure S2.** Calculated transmittance/reflectance of cross-CP and co-CP lights for the complementary MPM with *d*_1,2,3,4_ = 0 nm.

## Multipole interference of the non-complementary MPM


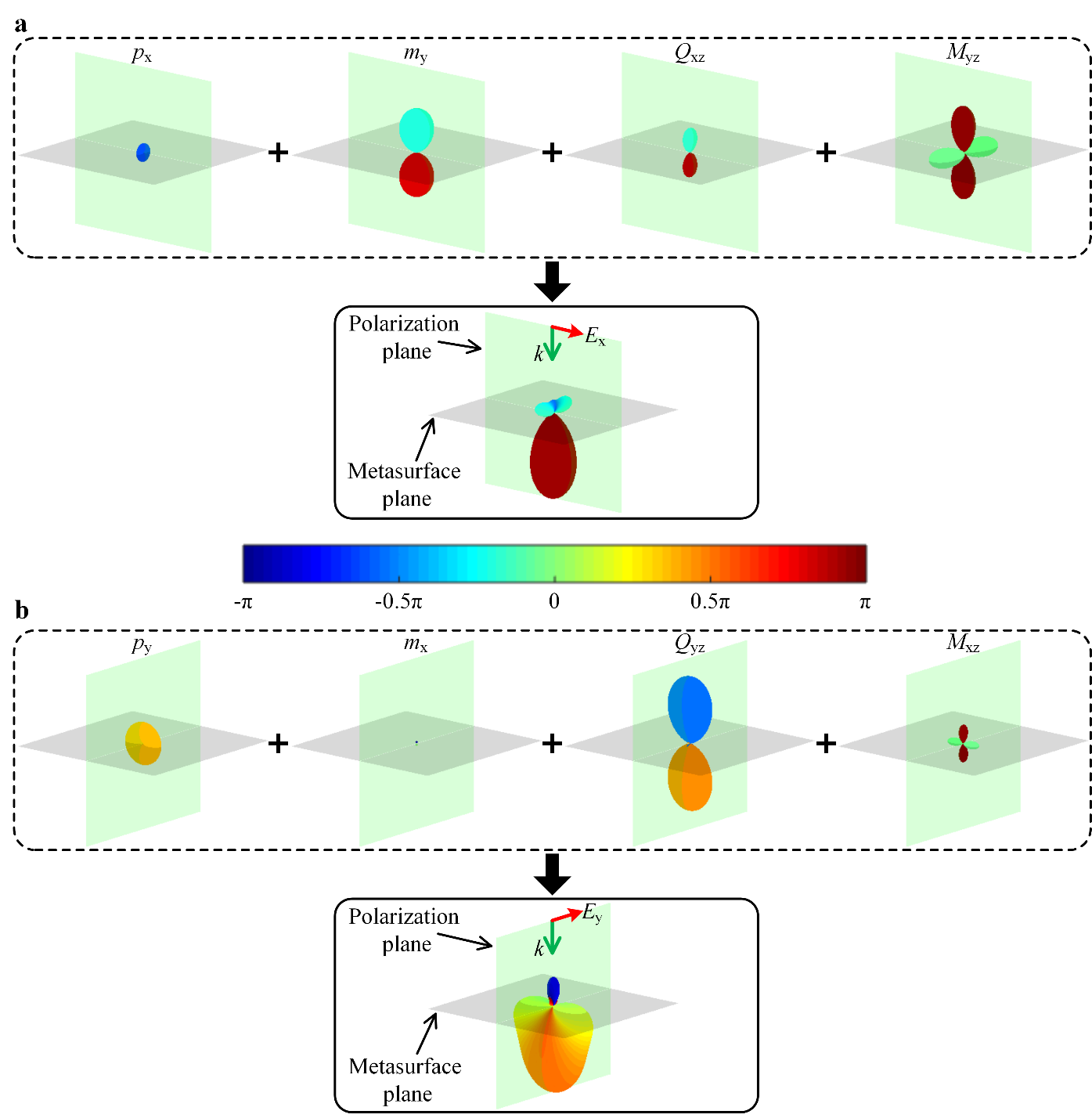


**Figure S3.** Far-field radiation patterns of each multipole in the non-complementary MPM with *d*_1,2,3,4_ = 10 nm and the resulted multipole interference patterns for two LPs at their reflection-minimum wavelengths, i.e. 705 nm for *x*-LP (**a**) and 780 nm for *y*-LP (**b**). The radiation patterns for each multipole are enlarged by 5 times in (**a**) and 2 times in (**b**). The colormap represents the phase of the radiated field.

## Suppression of reflection and absorption by non-complementary design


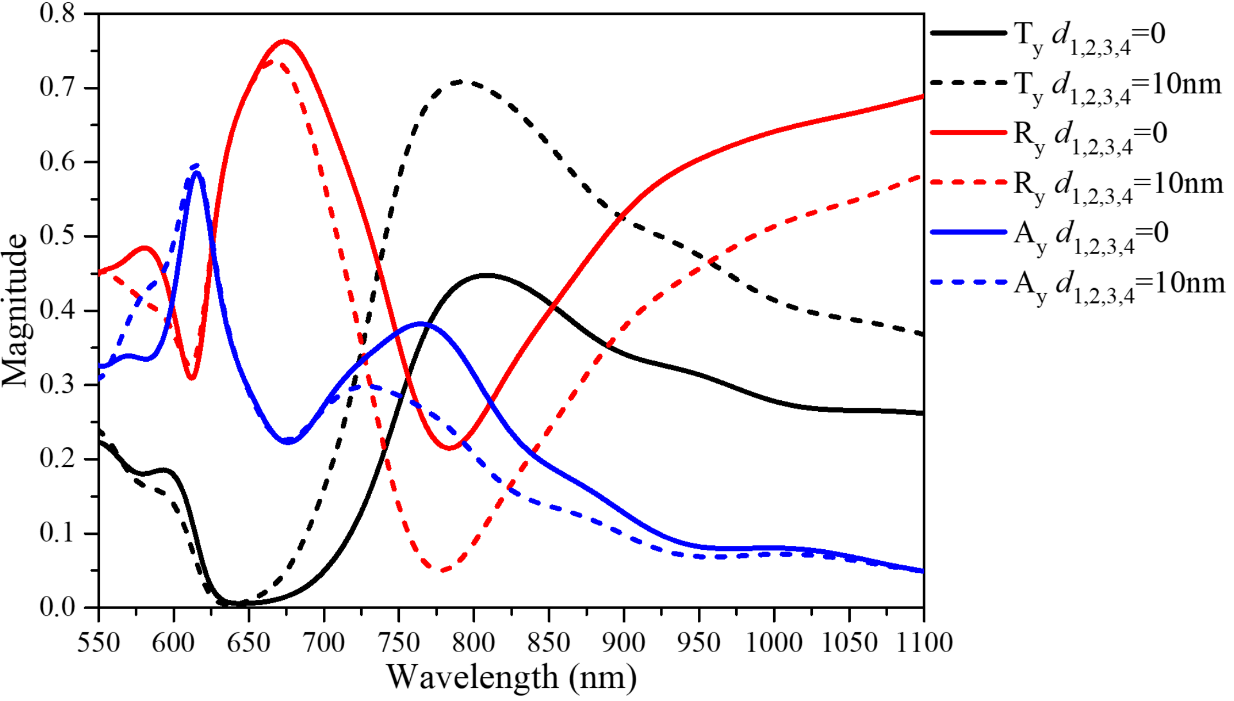


**Figure S4.** Calculated transmittance (*T*_y_), reflectance (*R*_y_) and absorption (*A*_y_) of complementary and non-complementary MPMs for *y*-LP input.

## Reduction of adjacent near-field coupling by non-complementary design


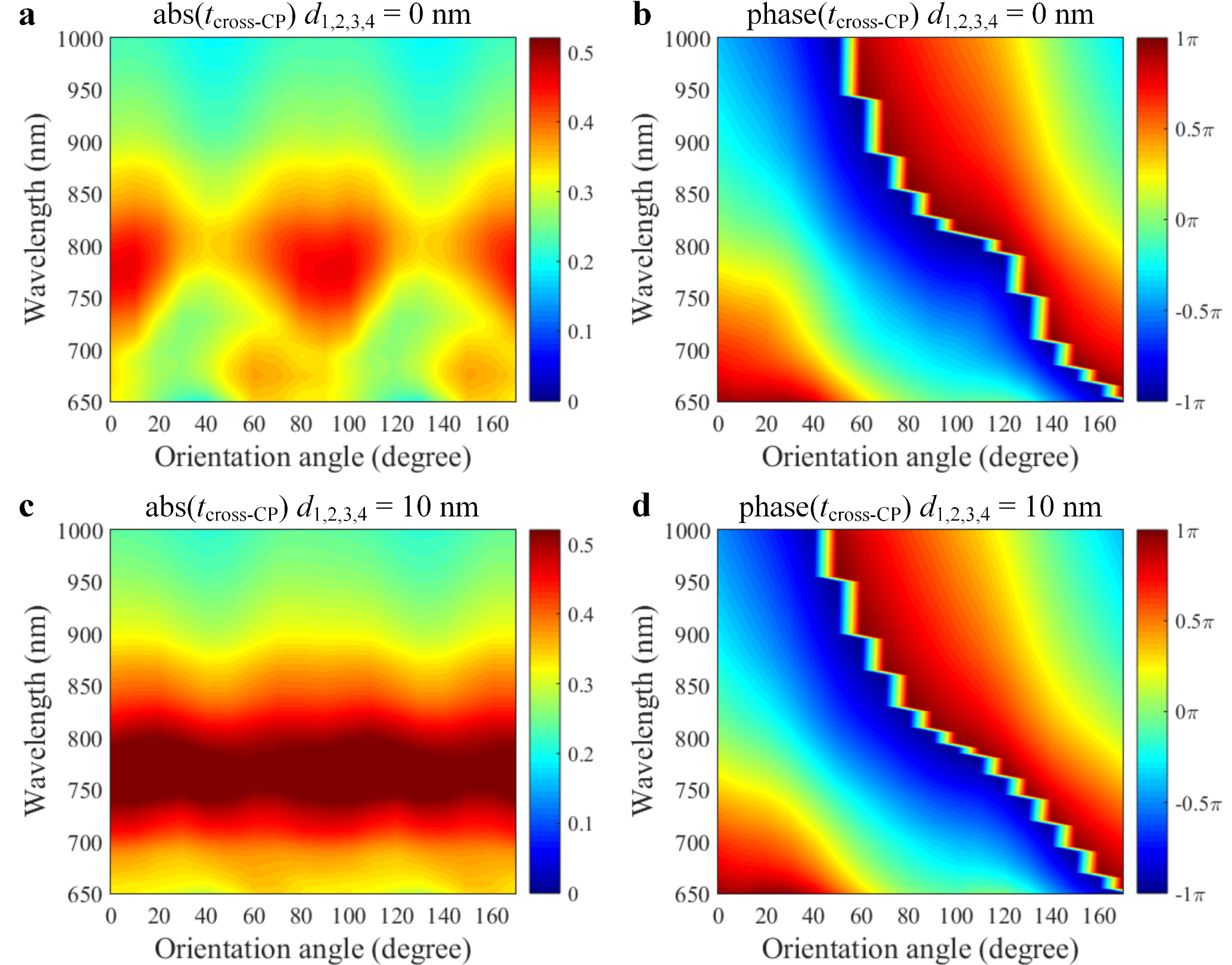


**Figure S5.** Calculated amplitude (**a**, **c**) and phase (**b**, **d**) of *t*_cross-CP_ as functions of the orientation angle and wavelength for the complementary (**a**, **b**) and non-complementary (**c**, **d**) meta-atoms. Both designs support good orientation-controlled phase responses in a broadband. However, the complementary meta-atom has fluctuant amplitudes with orientations, while the non-complementary meta-atom has higher and relatively flat amplitudes. This indicates the weaker near-field coupling of the non-complementary design.


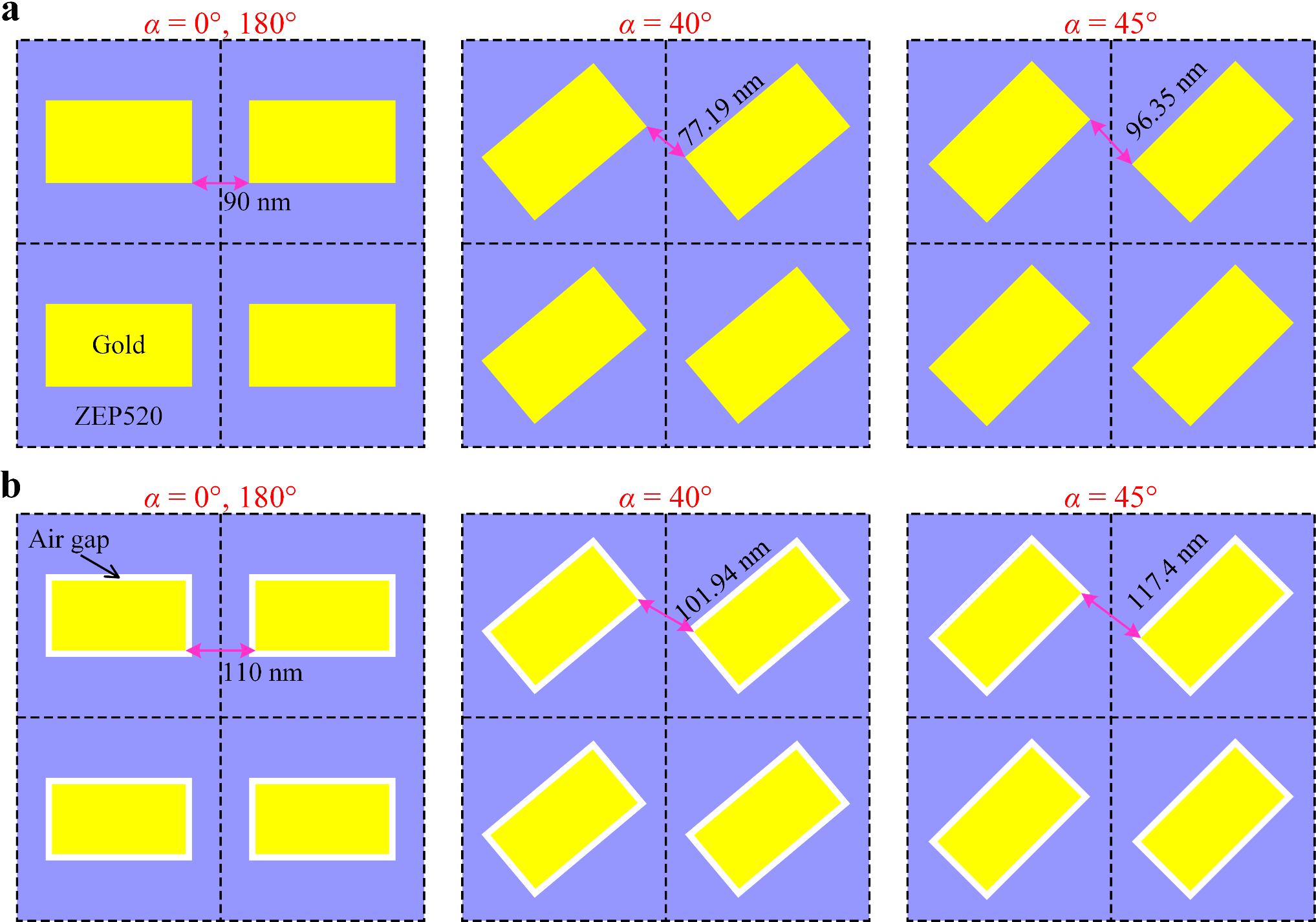


**Figure R6.** Schematic illustration of the arrangement of periodic nanorods at bottom layer for the complementary (**a**) and non-complementary (**b**) meta-atoms with various orientation angles and the gap distance between their nearest corners. The gap distance for *α* = 40° and 130° is smaller than the one for other angles. This means the adjacent coupling is stronger and thus the transmission amplitude is smaller, which agrees with our simulation in Fig. 2. In the non-complementary design, the nearest gap distance between two nanorods is increased. On the other hand, the field is confined into the air gap, which further reduces the near-field coupling.


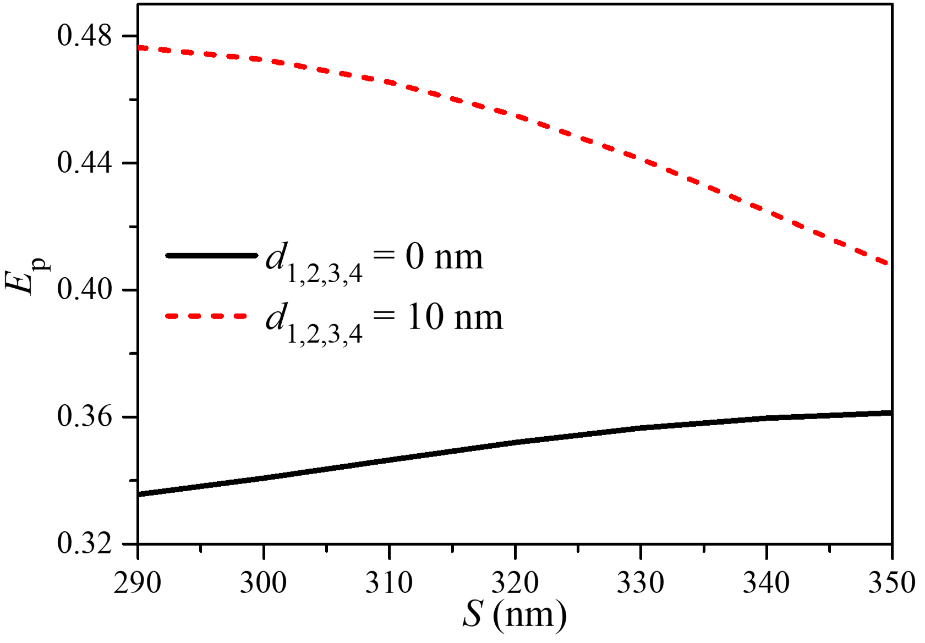


**Figure S7.** Calculated *E*_p_ as a function of the sub-period for the complementary and non-complementary meta-atoms.

## Relaxed fabrication tolerance


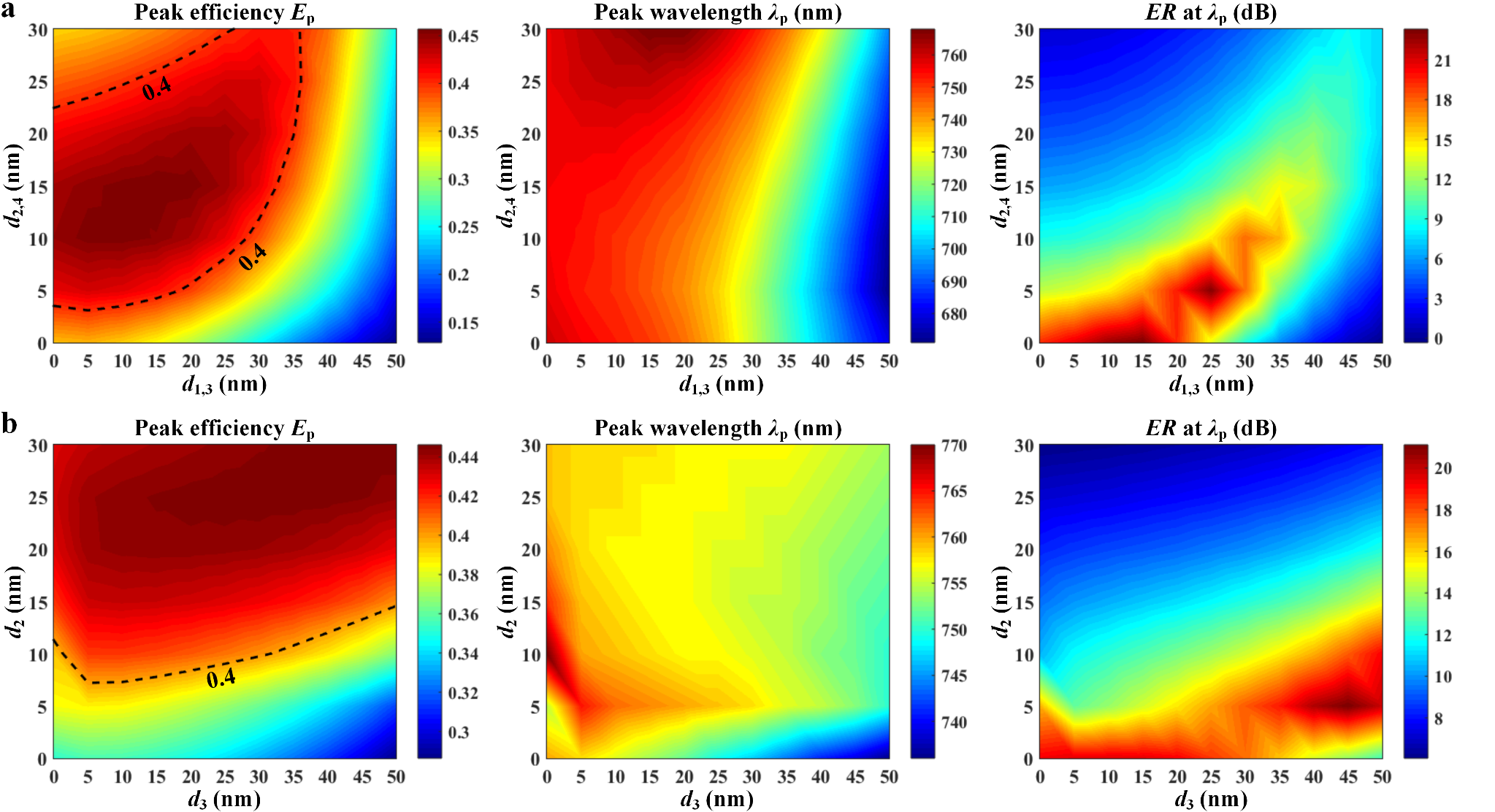


**Figure S8.** Calculated *E*_p_, *λ*_p_, and extinction ratio (ER) at *λ*_p_ of non-complementary MPMs as functions of *d*_1_ = *d*_3_ and *d*_2_ = *d*_4_ (**a**), and as functions of *d*_2_ and *d*_3_ when *d*_1,4_ = 0 (**b**). Other dimension parameters are fixed at *S* = 320 nm, *l* = 230 nm, *w* = 130 nm, *t* = 35 nm, and *t*_r_ = 180 nm. The black dashed lines mark out the contours of 0.4. For both the symmetric and asymmetric nanorod dimension mismatches, *E*_p_ overcomes 40% and ER exceeds 5 dB at visible wavelengths for a wide range of mismatching values.


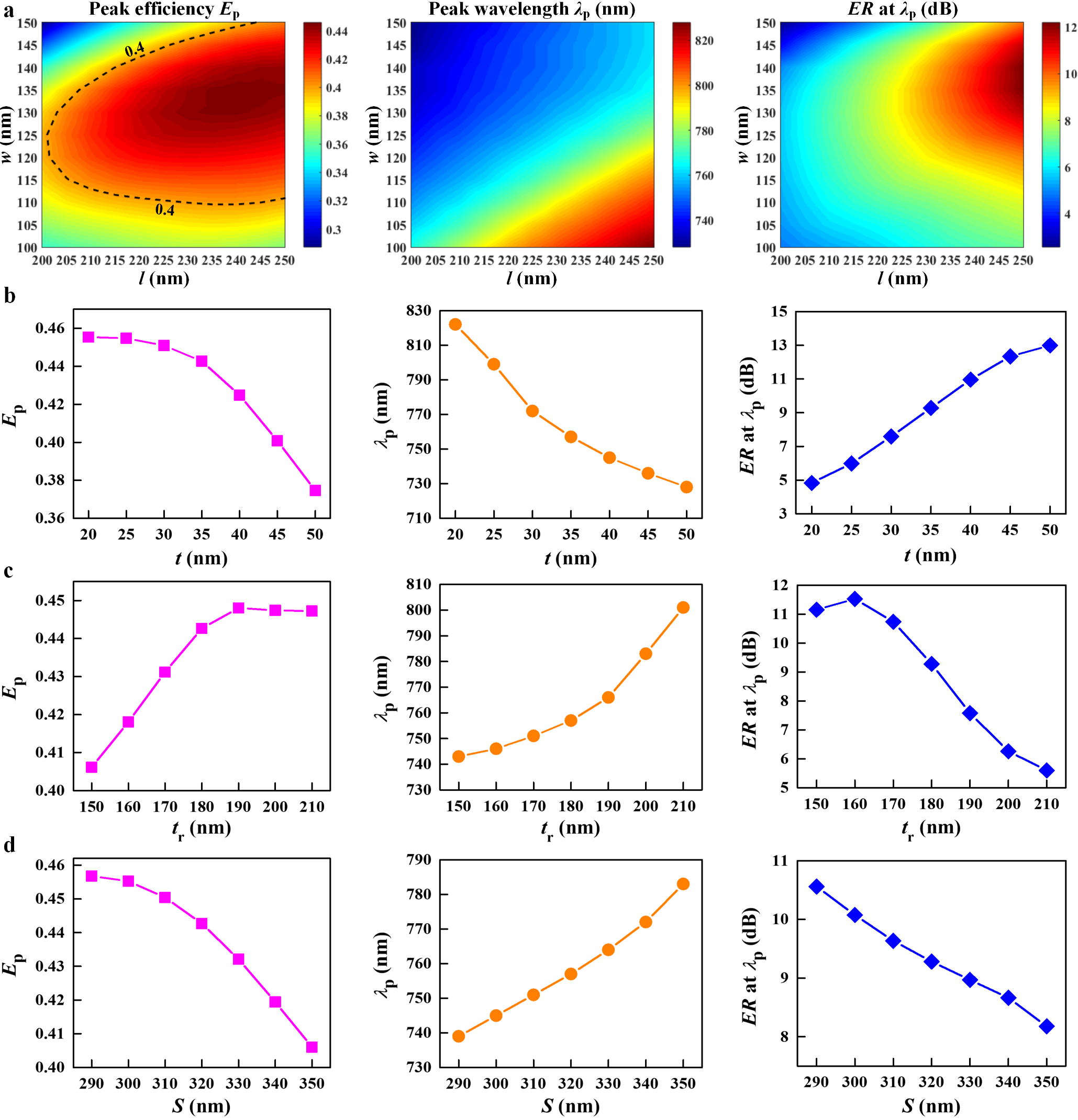


**Figure S9.** Calculated *E*_p_, *λ*_p_, and ER at *λ*_p_ of non-complementary MPMs as functions of *l* and *w* with *S* = 320 nm, *t* = 35 nm, and *t*_r_ = 180 nm (**a**), as a function of *t* with *S* = 320 nm, *l* = 230 nm, *w* = 130 nm, and *t*_r_ = 180 nm (**b**), as a function of *t*_r_ with *S* = 320 nm, *l* = 230 nm, *w* = 130 nm, and *t* = 35 nm (**c**), and as a function of *S* with *l* = 230 nm, *w* = 130 nm, *t*_m_ = 35 nm and *t*_r_ = 180 nm (**d**). In all simulations, *d*_1,4_ = 0 and *d*_2,3_ = 20 nm. *E*_p_ is larger than 40% and the corresponding ER exceeds 5 dB in a wide dimension space. These simulations in Fig. S6 and Fig. S7 indicate the fabrication tolerance of our proposed non-complementary MPM in realizing high performance.

## Multipole excitation of the asymmetric non-complementary MPM


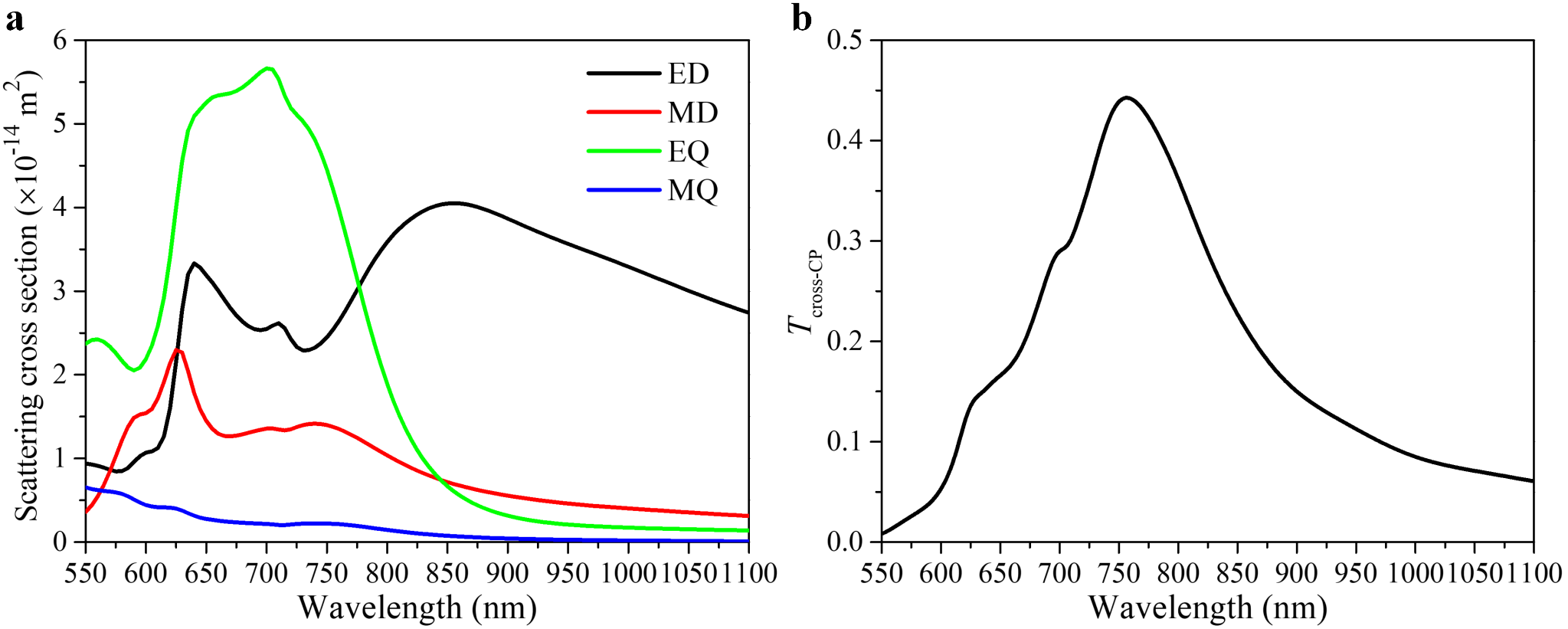


**Figure S10.** Calculated scattering cross sections (**a**) from each multipole and cross-CP transmittance (**b**) for the non-complementary MPM with *d*_1,4_ = 0 and *d*_2,3_ = 20 nm with a RCP input. The peak cross-CP transmittance is 44.3% at 757 nm.

## Experimental *T*_cross-CP_ with LCP input


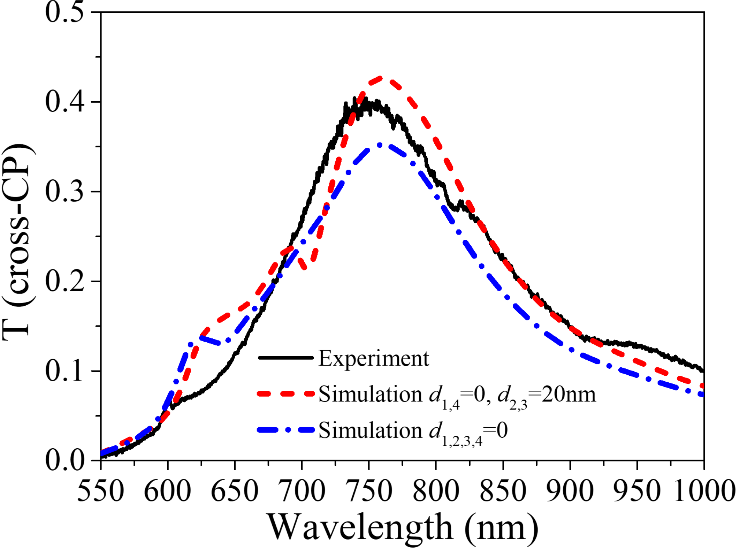


**Figure S11.** Measured cross-CP transmission efficiency of non-complementary MPM with LCP input and its comparison with simulations of MPMs with and without non-complementarities.

## High performance beam deflection of the demonstrated GM

Figure **S12a** illustrates the theoretical configuration of one unit cell of the non-complementary GM by applying dimension mismatch equations of and . The unit of the mismatches is nanometer. Simulation of this configuration results in peak efficiency of 36.6% for RCP input and 32.2% for LCP input, which are larger than that of complementary GM. The small difference resulting from RCP vs. LCP inputs is due to the asymmetry of the nanorod dimension mismatches. The experimental fabrication is slightly different from this theoretical configuration. In order to have a better prediction of the fabricated non-complementary GM, we measure the bottom mismatch values (i.e. *d*_1,2,3,4_) of each SU in the SEM images and take the average values. Figure **S12b** shows configuration of one unit cell of the fabricated GM with the measured averaged *d*_1,2,3,4_ values utilized in the simulation. Figure **S12c** and **S12d** are the amplitude and phase of the cross-CP transmission coefficients for SU1-SU8 with RCP and LCP inputs, respectively. The results are taken at 750 nm. First, each SU has larger amplitude responses than that of complementary one in Fig. **2e**. Second, both the amplitude and phase deviations are smaller than that of complementary one. In spite of the different mismatch values for each SU, the amplitude deviation is smaller than 0.04 and the phase deviation is smaller than 0.08π for both RCP and LCP inputs. These small amplitude and phase deviations are due to reducing the adjacent coupling from the non-complementary design. As a result, the theoretical efficiency of the demonstrated non-complementary GM (*E*_p_ = 40.4% for RCP and *E*_p_ = 38.3% for LCP) is only a few percent lower than the one of non-complementary regular MPM (*E*_p_ = 44.3% for RCP and *E*_p_ = 42.6% for LCP), and good quality of anomalous diffraction is supported, as shown in Fig. **S12(e-h)**. The reduced coupling between nanorods is further shown by the electric field distributions on the nanorod layer of one unit cell for both complimentary and non-complimentary GMs in Fig. **S12i**. For complementary design (Fig. **S12i-top**), the near-field coupling between adjacent SUs is strong. Conversely, the field is strongly localized within the introduced air gap (Fig. **S12i-bottom**) instead of the resist area between each SUs which result in weak adjacent coupling for the non-complementary GM.

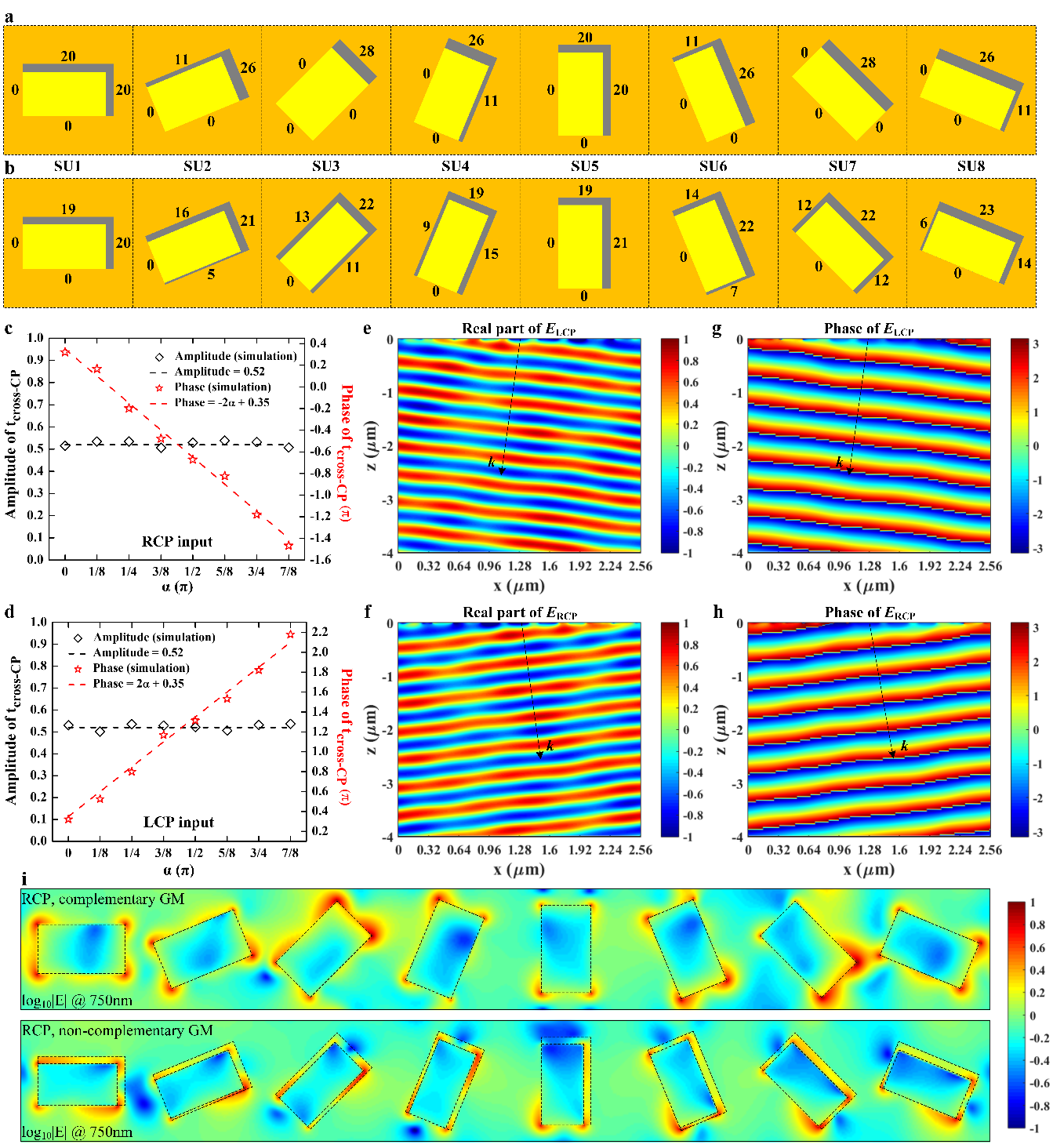


**Figure S12.** **a** Theoretical configuration of one unit cell of the non-complementary GM. **b** Configuration of one unit cell of the fabricated GM with averaged nanorod dimension mismatch values applied in simulation for each SU. **c-d** Amplitude and phase of the cross-CP transmission coefficients of SU1-SU8 with RCP (**c**) and LCP (**d**) inputs. **e-h** Real part (**e, f**) and phase (**g, h**) of the cross-CP electric fields in the propagation after the non-complementary GM for normal input along –*z* direction. **i** Log_10_|*E*| distributions on the nanorod layer of complementary and non-complementary GM. Reduced adjacent coupling is enabled in the non-complementary GM. All results are taken at 750 nm.

## Measurement of beam deflection with LCP input


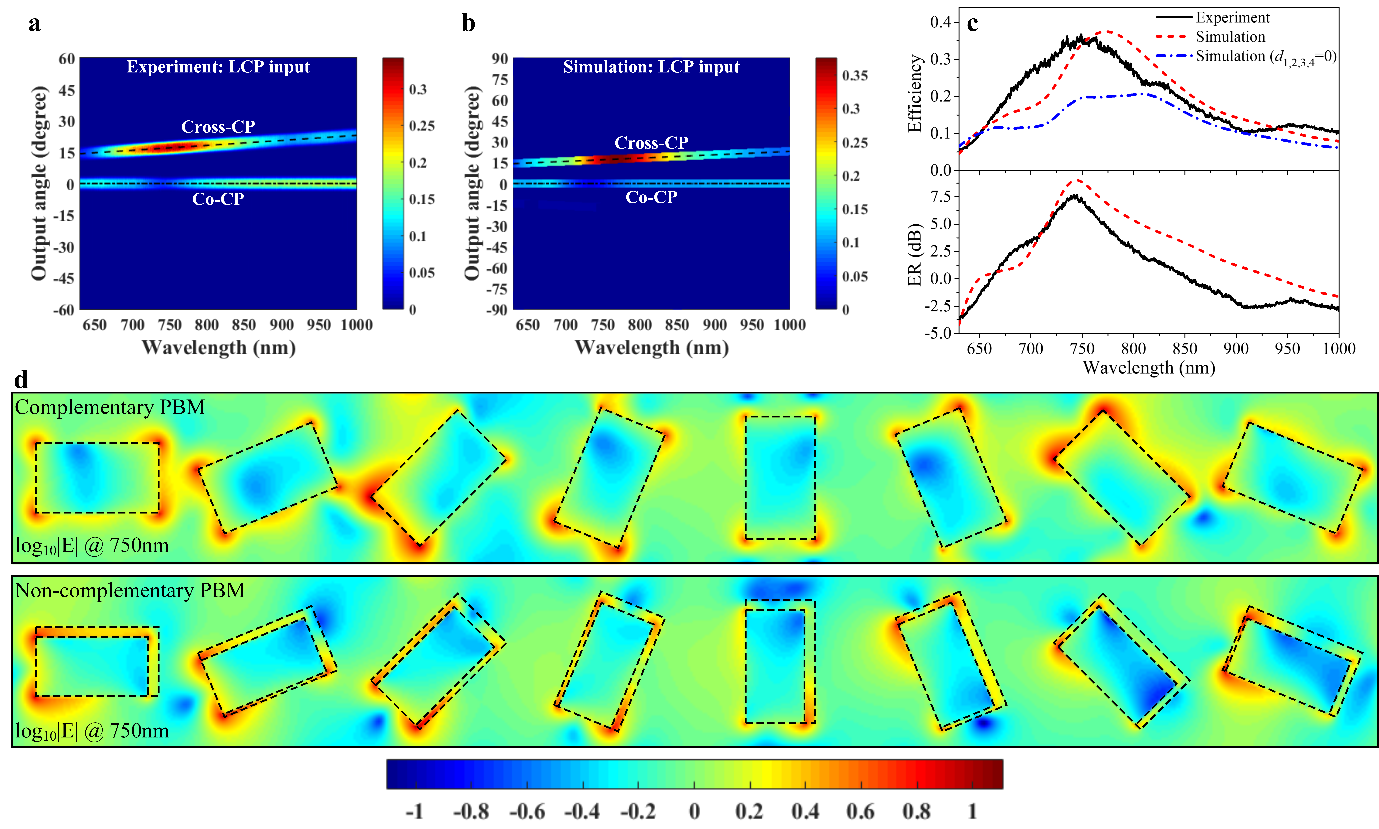


**Figure S13.** **a-b** Measured (**a**) and simulated (**b**) transmittance of the cross-CP and co-CP lights for non-complementary GM as functions of the wavelength and output angle for LCP input with normal incidence. The black dashed and dash-dotted lines represent the theoretical diffraction angle of the anomalous and normal diffractions, respectively. **c** Measured and simulated deflection efficiency of the anomalous beam and related ER over normal beam as a function of wavelength for LCP input. The solid, dashed, and dash-dotted lines represent the experimental results, simulation of GM with asymmetric mismatch values shown in Fig. **S12b**, and simulation of complementary GM, respectively. **d** Log_10_|*E*| distributions on the bottom layer of GM for LCP input.

## Diffraction to undesired high orders

According to the generalized Snell’s law, the output angle of diffraction into different orders is determined by^21^:

(S1)

For anomalous diffraction of cross-CP (+ for LCP input, - for RCP input), while for normal diffraction of co-CP . Thus, for normal incidence and transmitting into air, the diffraction equations for anomalous and normal diffractions are:

$\sin\theta_{t}^{a}=\frac{(m\pm1)\lambda}{\Lambda},\text{ }\sin\theta_{t}^{n}=\frac{m\lambda}{\Lambda}$ (S2)

Considering the period Λ = 2.56 μm and the shortest wavelength of 630 nm in our study, the m = -3 ~ 5 orders are supported for anomalous diffraction of RCP input and the m = -5 ~ 3 orders are supported for anomalous diffraction of LCP input. With respect to normal diffraction, the m = -4 ~ 4 orders exist. Figure **S14** and **S15** show the calculated diffraction efficiency into different orders with RCP and LCP inputs at normal incidence, respectively. Note that Fig. **S14** and **S15** are drawn based on the same simulation data in Fig. **5(c)** and Fig. **S13b**, except that here it is presented in the log scale in order to have a clearer view of the high orders with low efficiency. The black dashed lines are the theoretical output angles determined by Eq. (S2). We can see that the output angles of the simulation perfectly match with the theory. In addition, most of the power is diffracted into the zero order. The diffraction to high orders is negligible beyond 800 nm and smaller than one tenth of the zero order in the studied wavelength range.


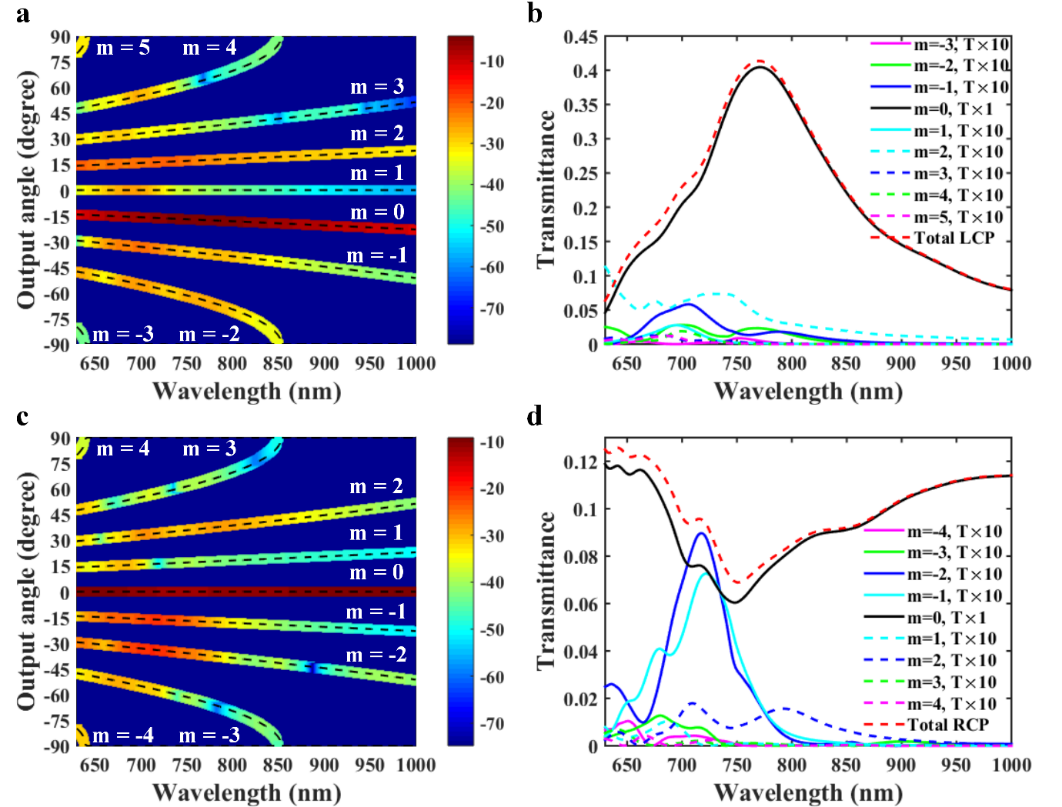


**Figure S14.** Calculated transmittance of the LCP (**a**) and RCP (**c**) lights in log scale as functions of output angle and wavelength for RCP input with normal incidence. The black dashed lines represent the theoretical diffraction angle of different orders in Eq. (S2). Transmittance of zero-order diffraction and magnified transmittance of high orders of LCP (**b**) and RCP (**d**).


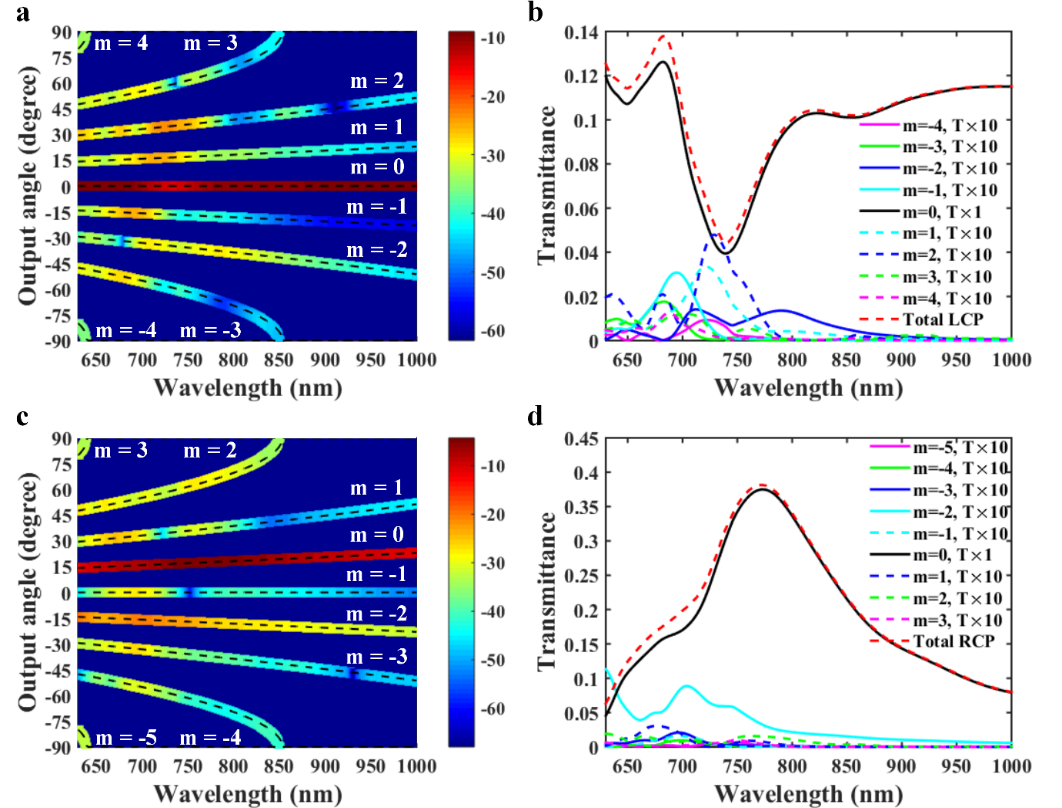


**Figure S15.** Calculated transmittance of the LCP (**a**) and RCP (**c**) lights in log scale as functions of output angle and wavelength for LCP input with normal incidence. The black dashed lines represent the theoretical diffraction angle of different orders in Eq. (S2). Transmittance of zero-order diffraction and magnified transmittance of high orders of LCP (**b**) and RCP (**d**).

## Beam deflection at various CW wavelengths


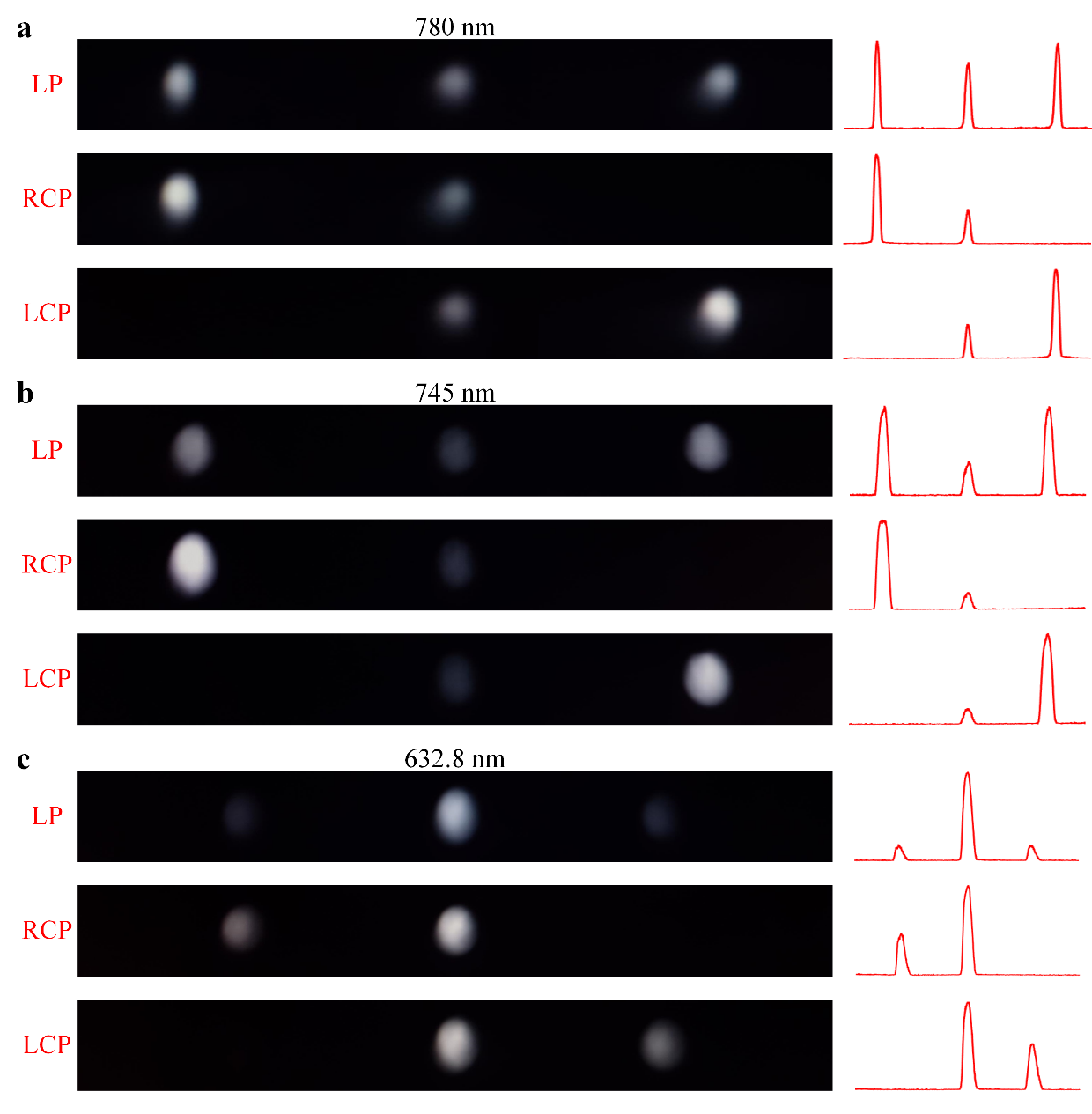


**Figure S16.** **a-c** Far-field diffraction patterns of the GM beam deflector at 780 nm (**a**), 745 nm (**b**), and 632.8 nm (**c**) for LP, RCP and LCP inputs. The figures on the right are the related normalized intensity profiles on a line that crosses the center of the light spots. For each wavelength, the total input power is the same for three inputs and adjusted so that there is no saturation on the camera.

## Holography images at various CW wavelengths


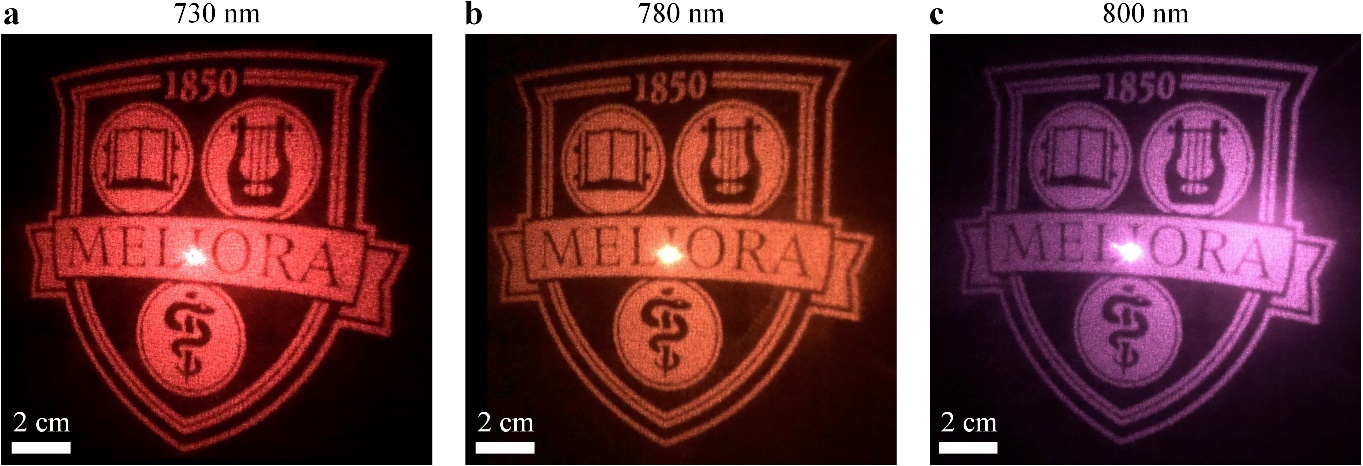


**Figure S17.** Experimental holography images at wavelengths of 730 nm (**a**), 780 nm (**b**) and 800 nm (**c**).

## Metasurface designed for shorter wavelength in the visible


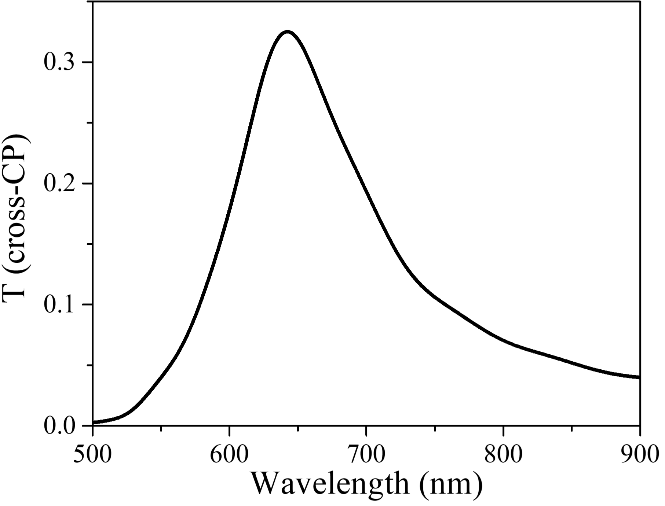


**Figure S18.** A metasurface design with optimized efficiency at shorter wavelength in the visible. The geometric dimension of this design is *S* = 220 nm, *l* = 150 nm, *w* = 85.5 nm, *t* = 43.1 nm, *t*_r_ = 147 nm, *d*_1,4_ = 0, and *d*_2,3_ = 20 nm. The maximum simulation efficiency is 33% at 642 nm.

## Measurement setups


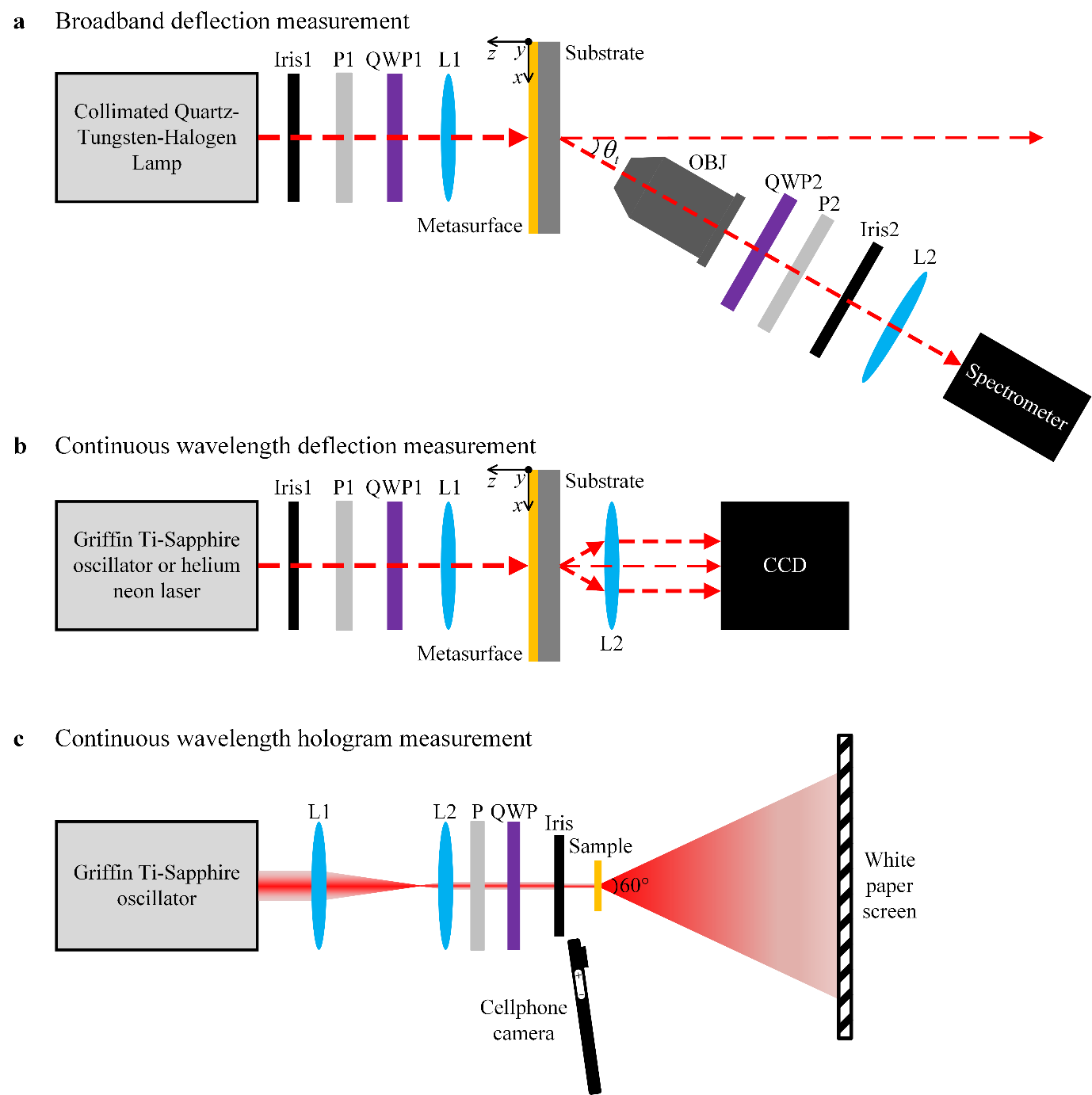


**Figure S19.** Schematics of the experimental setups for broadband deflection (**a**), CW deflection (**b**), and CW hologram (**c**) measurements. P: polarizer, QWP: quarter wave plate, L: lens, OBJ: objective, CCD: charged coupled device.

## References

1. Genevet, P. *et al*, Recent advances in planar optics: from plasmonic to dielectric metasurfaces. *Optica* **4**, 139-152 (2017).
2. He, Q. *et al*, High-Efficiency Metasurfaces: Principles, Realizations, and Applications. *Advanced Optical Materials* **6**, 1800415 (2018).
3. Gramotnev, D. K. & Bozhevolnyi, S. I. Plasmonics beyond the diffraction limit. *Nature Photonics* **4**, 83-91 (2010).
4. Stockman, M. I. Nanoplasmonics: past, present, and glimpse into future. *Optics Express* **19**, 22029-22106 (2011).
5. Ekmel, O. Plasmonics: Merging Photonics and Electronics at Nanoscale Dimensions. *Science* **311** 189-193 (2006).
6. Stewart, M. E. *et al*, Nanostructured Plasmonic Sensors. *Chemical Reviews* **108**, 494-521 (2008).
7. Kauranen, M. & Zayats, A. V. Nonlinear plasmonics. *Nature Photonics* **6**, 737-748 (2012).
8. Xie, Z. H. *et al*, Plasmonic Nanolithography: A Review. *Plasmonics* **6**, 565 (2011).
9. Pelton, M. Modified spontaneous emission in nanophotonic structures. *Nature Photonics* **9**, 427-435 (2015).
10. Kabashin, A. V. *et al*, Plasmonic nanorod metamaterials for biosensing. *Nature Materials* **8**, 867-871 (2009).
11. Wu, C. *et al*, Fano-resonant asymmetric metamaterials for ultrasensitive spectroscopy and identification of molecular monolayers. *Nature Materials* **11**, 69-75 (2011).
12. Kravets, V. G. *et al*, Singular phase nano-optics in plasmonic metamaterials for label-free single-molecule detection. *Nature Materials* **12**, 304-309 (2013).
13. Lee, J. *et al*, Giant nonlinear response from plasmonic metasurfaces coupled to intersubband transitions. *Nature* **511**, 65-69 (2014).
14. Alam, M. Z., De Leon, I. & Boyd, R. W. Large optical nonlinearity of indium tin oxide in its epsilon-near-zero region. *Science* **352**, 795-797 (2016).
15. Alam, M. Z. *et al*, Large optical nonlinearity of nanoantennas coupled to an epsilon-near-zero material. *Nature Photonics* **12**, 79-83 (2018).
16. Brinks, D. *et al*, Plasmonic antennas as design elements for coherent ultrafast nanophotonics. *Proceedings of the National Academy of Sciences* **110**, 18386-18390 (2013).
17. Guo, P. J. *et al*, Ultrafast switching of tunable infrared plasmons in indium tin oxide nanorod arrays with large absolute amplitude. *Nature Photonics* **10**, 267-273 (2016).
18. Shcherbakov, M. R. *et al*, Ultrafast all-optical tuning of direct-gap semiconductor metasurfaces. *Nature Communications* **8**, 17 (2017).
19. Shcherbakov, M. R. *et al*, Ultrafast All-Optical Switching with Magnetic Resonances in Nonlinear Dielectric Nanostructures. *Nano Letters* **15**, 6985-6990 (2015).
20. Akselrod, G. M. *et al*, Probing the mechanisms of large Purcell enhancement in plasmonic nanoantennas. *Nature Photonics* **8**, 835-840 (2014).
21. Yu, N. F. *et al*, Light Propagation with Phase Discontinuities: Generalized Laws of Reflection and Refraction. *Science* **334**, 333-337 (2011).
